# Chromosomal Density of Cancer Up-Regulated Genes, Aberrant Enhancer Activity and Cancer Fitness Genes Are Associated with Transcriptional Cis-Effects of Broad Copy Number Gains in Colorectal Cancer

**DOI:** 10.3390/ijms20184652

**Published:** 2019-09-19

**Authors:** Daniele Filippo Condorelli, Anna Provvidenza Privitera, Vincenza Barresi

**Affiliations:** Department of Biomedical and Biotechnological Sciences, Section of Medical Biochemistry, University of Catania, 95123 Catania, Italy; anna.privitera@studium.unict.it

**Keywords:** cancer aneuploidy, gene copy number abnormalities, gene-dosage effect, colorectal cancer, enhancer, cancer fitness

## Abstract

Broad Copy Number Gains (BCNGs) are copy-number increases of chromosomes or large segments of chromosomal arms. Publicly-available single-nucleotide polymorphism (SNP) array and RNA-Seq data of colon adenocarcinoma (COAD) samples from The Cancer Genome Atlas (TCGA) consortium allowed us to design better control groups in order to identify changes in expression due to highly recurrent BCNGs (in chromosomes 20, 8, 7, 13). We identified: (1) *Overexpressed Transcripts* (*OverT*), transcripts whose expression increases in “COAD groups bearing a specific BCNG” in comparison to “control COAD groups” not bearing it, and (2) *up-regulated/down-regulated transcripts*, transcripts whose expression increases/decreases in COAD groups in comparison to normal colon tissue. An analysis of gene expression reveals a correlation between the density of up-regulated genes per selected chromosome and the recurrence rate of their BCNGs. We report an enrichment of gained enhancer activity and of cancer fitness genes among OverT genes. These results support the hypothesis that the chromosomal density of overexpressed cancer fitness genes might play a significant role in the selection of gained chromosomes during cancer evolution. Analysis of functional pathways associated with OverT suggest that some multi-subunit protein complexes (eIF2, eIF3, CSTF and CPSF) are candidate targets for silencing transcriptional therapy.

## 1. Introduction

In the context of the theory of carcinogenesis as an evolutionary process, a high endogenous mutagenic rate (genome instability) will generate both selectable pathogenic mutations (driver mutations) and neutral mutations (passenger mutations) that are randomly associated with pathogenic mutations during cancer evolution. One of the major issues in cancer research is the ability to differentiate driver from passenger mutations. Several criteria have been used in the case of small-scale intragenic mutations (point mutations and short indels), including their recurrence frequency in different cancer samples, and their functional and clinical consequences [1,2]. Similar criteria have been applied to some types of chromosomal aberrations, such as gene fusions at the breakpoint of chromosomal translocations, or focal deletions or amplifications of specific genes [3]. However, much less is known about broad low-level chromosomal gains, such as the trisomy or tetrasomy of whole chromosomes or chromosomal arms.

Such chromosomal abnormalities, a form of aneuploidy, can be also called broad (or arm-level) copy number aberrations of the gain type or simply broad copy number gains (BCNGs). In this case the single mutational event is the increased chromosome copy number (due, for instance, to a single chromosomal mis-segregation event), and the genes located in the BCNG region are a large number ranging from hundreds to several thousands. The point of view is shifting from a single mutation-single candidate pathogenic gene to single mutation-multiple candidate pathogenic genes [4]. In this context it is conceivable that only a subgroup of genes located in BCNGs are indeed acting as driver genes, while the remaining genes can be considered as passenger ones (affected by the same mutation, but not taking part in determining the selectable phenotypic change). 

Evidence that some specific BCNGs may act as driver mutations is mainly based on their rather high recurrence frequency in both chromosomally stable and unstable cancers, on their cancer-type specificity, or on the results of experimental chromosome transfer [5,6,7,8,9,10]. However, very little is known about driver genes or gene networks associated with BCNGs that can provide a selectable advantage to cancer cells. The potentially large number of involved genes, and the fact that each gene can individually provide only a weak effect, make their identification extremely difficult [4,11].

An obvious mechanism linking BCNGs to selectable phenotypic effects is the so called “gene-dosage transcriptional effect”, a gene copy-number-dependent transcriptional effect. Along this line of reasoning the presence of a “gene-dosage transcriptional effect” is only a pre-requisite to identify potential driver genes associated with this type of chromosomal aberrations. Indeed, the transcriptional effect does not translate necessarily into selectable cancer advantageous features [12,13]. Therefore, BCNG-associated driver genes can represent a subgroup of genes susceptible to a gene-dosage transcriptional effect. With this in mind, a deeper knowledge on factors influencing the gene-dosage transcriptional effect is a crucial step in this research field. It has been suggested that the vast majority of genes are dosage sensitive, and that the gene-dosage transcriptional effect is correlated to a gene copy number (CN), so that a CN equal to three would produce, on average, a 1.2–1.3 fold-increase of transcription [5,6,7,14,15,16,17,18]. However, the sensitivity to this effect is not equal for all genes [5,14] and might depend on the cellular context. Moreover, the transcriptional response to copy number changes is not necessarily linear. Indeed, other upstream factors, such as the level of specific transcription factors or chromatin accessibility, may represent a rate-limiting step for transcription, and such factors may depend on the specific cellular state.

We have recently reported that genes whose expression is up-regulated in colon cancer cells (in comparison to normal tissue) are more susceptible to the gene-dosage transcriptional effects in comparison to downregulated genes [19]. In the present paper we extend our previous results analysing data on colon cancer samples provided by The Cancer Genome Atlas (TCGA) consortium, showing that the chromosomal density of cancer up-regulated genes in selected aberrant chromosomes is correlated to the frequencies of BCNGs in the same chromosomes. Moreover, we analyse the effects on protein-coding and non-coding transcripts, and evaluate the possible correlation between aberrant gene enhancer hyperactivity and BCNG-associated transcriptional effects. Finally, we took advantage of the availability of studies by high-resolution CRISPR screens that have defined a cancer fitness gene as any gene whose knockdown decreases cell growth and proliferation in cancer cell lines [20], and sought to establish the relationship between cancer fitness properties and BCNG transcriptional dysregulation.

## 2. Results

### 2.1. Chromosomal Distribution of Arm-Level Copy Number Abnormalities in TCGA COAD Samples

**Figure 1:**
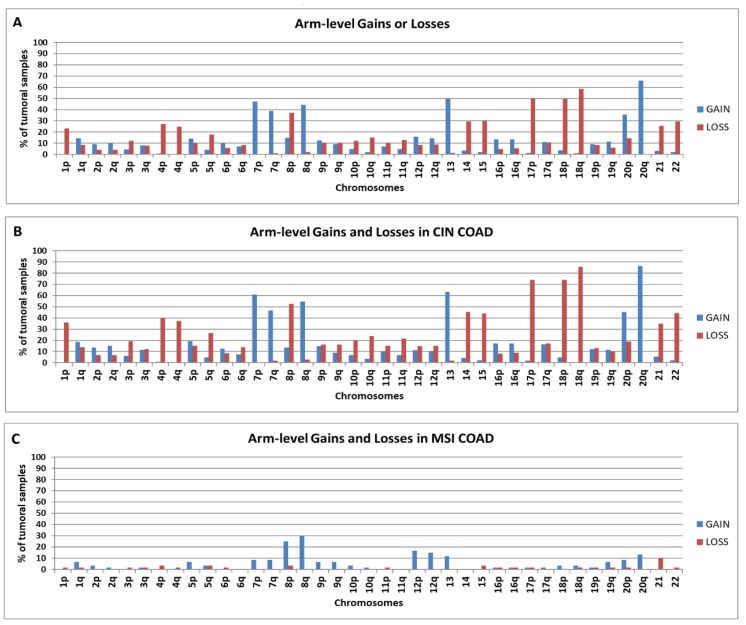
Results are expressed as the percentage of tumour samples bearing arm-level gains or losses in the p or q arm in all colon adenocarcinoma (COAD) samples (**A**), in Chromosomal INstability colon adenocarcinoma (CIN COAD) samples (**B**) or MicroSatellite Instability (MSI) COAD samples (**C**).

Among Copy Number Abnormalities (CNAs), we selected arm-level CNAs defined as somatic chromosomal aberrations that involve more than 50% of a chromosomal arm (p or q). In Figure 1A we report the chromosomal distribution of arm-level gains and losses in the examined population of 433 colon adenocarcinoma (COAD) samples (data obtained by Affymetrix SNP 6.0 array and downloaded from GDC Data Portal, NIH, National Cancer Institute, https://portal.gdc.cancer.gov/). 

In agreement with previous data in colorectal cancer [21] a high frequency (> 20% of the analysed samples) of arm-level gains can be observed in chromosomes 20p/q, 13, 7p/q, 8q, and a high frequency of arm-level losses in 18p/q, 8p, 17p, 14, 15, 4p/q, 21, 22, 1p (Figure 1A). In several cases p-arm loss of a chromosome, such as chromosome 8 or 20, was associated with a q-arm gain of the same chromosome. This kind of alteration is remindful of a type of cytogenetic abnormality, called “isochromosome”, which is characterized from the loss of a chromosomal arm accompanied by the duplication of the remaining one, and is abbreviated as i(chromosome number and duplicated arm). Indeed, isochromosomes have frequently been cytogenetically detected in human cancer [22]. Although data on copy number changes do not formally prove the presence of an isochromosome, in the present paper we indicate as “isochromosome” any simultaneous loss of a chromosome arm associated with the gain of the remaining one. According to such an understanding, we calculate that i(8q) is present in 24.2% of COAD samples, i(20q) in 13.4%, i(17q) in 8.7%, i(1q) in 6.7%, i(5p) in 4.6%.

Moreover, we separately analysed 223 samples showing Chromosomal INstability (CIN) and 60 samples showing MicroSatellite Instability (MSI), using the clinical information provided by cBioPortal (www.cbioportal.org) [23,24] according to the definitions offered by Liu et al. [25]. Results on CIN samples (Figure 1B) are similar to those observed in the entire group of COAD samples with an obvious increase of the frequencies due to the higher number of arm-level CNAs per sample. Quantitative and qualitative differences can be observed in MSI samples (Figure 1C) in comparison to CIN ones. 

The decreased frequency of arm-level CNAs is in agreement with the well-known observation that the majority of MSI colorectal samples show a nearly normal karyotype [21], and references herein cited. Indeed, in the present study, 26 MSI tumours out of 60 (43.33%) do not bear any broad CNAs, and are considered as normal karyotype tumours. Moreover, other 15 MSI samples bear less than or equal to two arm-level CNAs, and are considered as a near-normal karyotype. In total, 68% of MSI tumours show a normal or a near-normal karyotype. In contrast, only 2% of the 223 CIN tumours show less than or equal to two broad CNAs. In MSI samples, arm-level gains in 8p/q are the most frequent chromosomal CNAs, while 18q losses, one of the most frequent aberrations in CIN COAD samples, are almost absent. 

### 2.2. Differential Expression Analysis of RNA-Seq Data in TCGA COAD Samples

**Figure 2:**
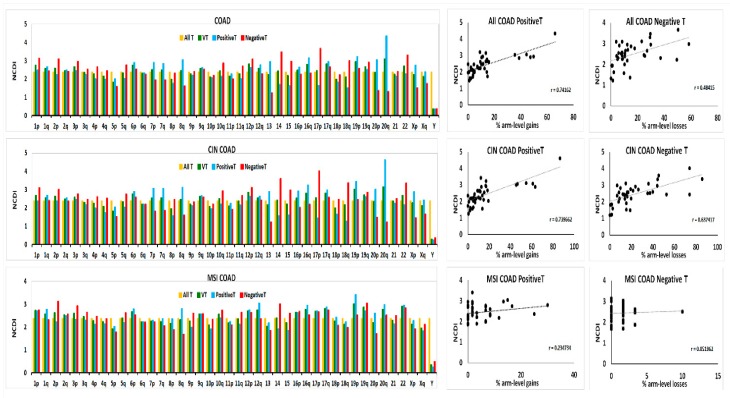
Percentage chromosomal distribution, expressed as NCDI (Normalized Chromosomal Distribution Index—see formula in Materials and Methods), of chromosomal density (number of specific transcripts divided by the total number of transcripts in that chromosome or chromosomal arm) of Variable Transcripts (VT), PositiveT and NegativeT. Values obtained for all transcripts (All T) located in a chromosome or chromosomal arm are also reported for comparison. Results obtained in COAD, CIN COAD and MSI COAD samples are shown in the left panels. Correlation plots of the NCDI values of PositiveT and NegativeT with the frequencies of arm-level gains and losses in each sample group are reported in the right panels. The Pearson’s r index is reported in each correlation plot.

Analysis of differential expression of transcript levels between all COAD samples and 41 normal colon tissues was performed by edgeR package [26,27] and expressed as Fold-Change (FC) in the comparison tumour vs. normal. We denominated Variable Transcripts (VT) those transcripts showing statistically significant changes, at a False Discovery Rate (FDR) adjusted *p*-value < 0.05. We called positive transcripts (PositiveT) and negative transcripts (NegativeT) those VT having FC > 1 or FC < 1, respectively. The chromosomal distribution of the up-regulated transcripts (PositiveT) and downregulated transcripts (NegativeT) are reported in Figure 2 (top panels), using the so-called Normalized Chromosomal Distribution Index (NCDI), as previously defined in Condorelli et al. [19]. 

Interestingly, chromosomes that bear frequently arm-level gains (BCNGs), such as Chr20, 8q, 7, and 13 show high NCDI values of PositiveT, reflecting a relatively high chromosomal density of up-regulated transcripts (number of up-regulated transcripts divided by the total number of transcripts in that chromosomal region). On the other hand, chromosomes showing a high frequency of arm-level losses show high NCDI values of NegativeT, indicating a relatively high chromosomal density of downregulated transcripts. This is confirmed by a high Pearson’s r correlation index between the NCDI values of PositiveT and the percentage of arm-level gains, or between NCDI values of NegativeT and the percentage of arm-level losses (Figure 2 top panels). However, it should be noted that 19p has a relatively high NCDI value of PositiveT, although bearing one of the lowest percentages of BCNGs (Figure 1 and Figure 2). 

Similar results have been obtained analysing 223 CIN COAD samples (Figure 2 middle panels). On the basis of these results, it is not possible to establish if the high chromosomal density of up-regulated transcripts is a simple consequence of the high frequencies of BCNGs in the selected chromosomes, or also whether or not it is an intrinsic property of the selected chromosomes in cancer, even when they are not copy-number aberrant (for this point, see the following Section 2.3 and Section 2.4). On the contrary, no significant correlation of PositiveT or NegativeT with arm-level chromosomal aberrations is observed by analysing 60 MSI samples, in accordance with the fact that those tumours show an almost normal karyotype (Figure 2 bottom panels). 

### 2.3. Chromosomal Distribution of Arm-Level CNAs in “Selected COAD” Groups

**Figure 3:**
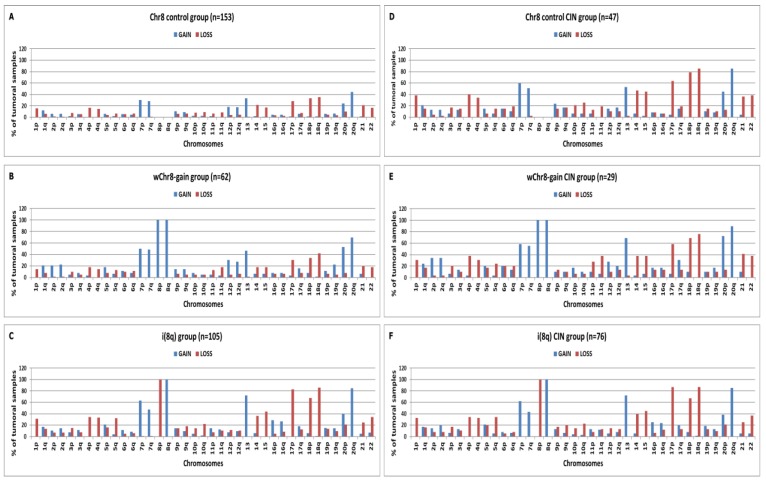
Arm-levels gains and losses in Chr8 control COAD group (**A**,**D**) and in wChr8-gain (**B**,**E**) and i(8q) (**C**,**F**) selected COAD groups. Results are expressed as a percentage of tumour samples bearing arm-level gains or losses in the p or q arm in all COAD samples (**A**–**C**) or in CIN COAD samples (**D**–**F**).

We organized COAD samples in groups, called “Selected COAD” groups, bearing a specific BCNG. Each “Selected COAD group” was compared with a corresponding “Control COAD” group composed of tumours lacking any arm-level CNAs on the chosen chromosome. Among all 433 COAD samples we selected groups characterized by the following BCNGs: whole (w) Chr20-gain, i(20q), wChr8-gain, i(8q), wChr13-gain and wChr7-gain. Similar groups were also organized for CIN COAD samples (*n* = 223). In Figure 3 the chromosomal distribution of arm-level gains and losses is shown for wChr8-gain and i(8q) in all tumour samples or in CIN samples. Results for the other groups are reported in Supplementary Figure S1. Since the “Chr8 Control group” (COAD samples without aberrations in Chr8, *n* = 153) contains many normal karyotype samples (such as MSI samples), the frequencies of chromosomal aberrations are very low (Figure 3A). On the other hand, the “Chr8 Control group” in CIN COAD samples (Chr8 control CIN group, *n* = 47, Figure 3D) shows higher frequencies of chromosomal aberrations with a profile similar to that observed in the wChr8-gain CIN group (except for Chr8 aberrations) (Figure 3E). In order to provide a quantitative score of the “profile similarity” between “control CIN groups” and “selected CIN groups”, we performed a correlation analysis between the frequencies of arm-level gains and losses in each chromosomal arms of the two different groups (obviously excluding the value of the frequency of gains in the selected chromosome of each group, since these values are 0% in the control group and 100% in the gain group). We obtained the following Pearson’s correlation coefficients: wChr20-gain vs. Chr20 control r = 0.83, i(20q) vs. Chr20 control r = 0.75, wChr8-gain vs. Chr8 control r = 0.92, i(8q) vs. Chr8 control r = 0.93, wChr7-gain vs. Chr7 control r = 0.96, wChr13-gain vs. Ch13 control r = 0.96. The complete correlation plots are reported in Supplementary Figure S1.

### 2.4. Transcriptome Analysis in “Selected COAD” Groups

For each COAD group, four different “indices” were used in order to estimate transcript level differences [19]: (a) Linear fold-changes obtained comparing all COAD samples (Selected + Control COAD) vs. normal colonic mucosae (denominated FC1); (b) linear fold-changes obtained comparing COAD samples bearing a specific arm-level CNA (Selected COAD group) to samples not bearing it (Control COAD group) (denominated FC2); (c) linear fold-changes obtained comparing “Control COAD” group to normal colonic mucosae (denominated FC3); (d) linear fold-changes obtained comparing “Selected COAD” group to normal colonic mucosae (denominated FC4). The number of patients in each “Selected COAD” and corresponding “Control COAD” group are reported in Figure 3 and Supplementary Figure S1.

We called OverT the VT expressing FC2 > 1.3, representing the transcripts that are overexpressed in the “selected COAD group” in comparison to the corresponding “control COAD group”. Finally, we called PositiveT those VT having FC3 > 1 and FC4 > 1, and NegativeT those VT expressing FC3 < 1 and FC4 < 1. In other words, PositiveT and NegativeT are transcripts that are up-regulated or downregulated, respectively, in both the “selected COAD group” and the “control COAD group”, in comparison to normal colon tissue. 

**Figure 4:**
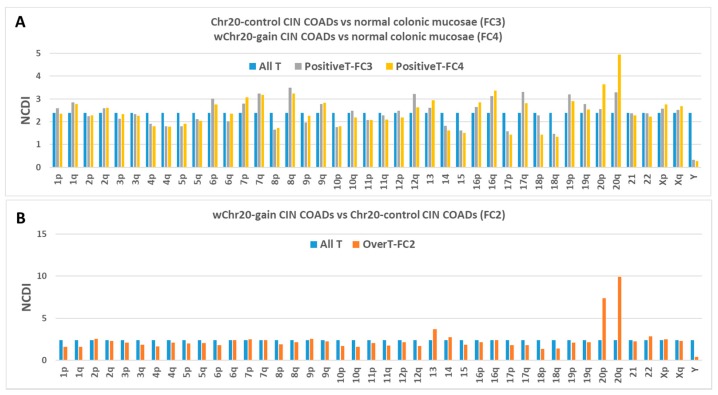
Percentage chromosomal distribution (expressed as NCDI) of different transcript classes in Chr20 control and wChr20-gain CIN-positive colon cancer groups: (**A**) All transcripts (All T), PositiveT-FC3, and PositiveT-FC4); (**B**) All T and OverT-FC2.

However, in the results shown in Figure 4A, PositiveT have been further subdivided in two categories: PositiveT-FC3 and PositiveT-FC4. PositiveT-FC3 represent transcripts up-regulated in the “control COAD group” in comparison to normal colon tissue (T having FC3 > 1 and FDR < 0.05 in the comparison “Control COADs” vs. normal colonic mucosae), while PositiveT-FC4 are transcripts up-regulated in “selected COAD group” (T having FC4 > 1 and FDR < 0.05 in the comparison “Selected COADs” vs. normal colonic mucosae). We calculated the percentage distribution of PositiveT-FC3 and PositiveT-FC4, expressed as NCDI values, for each chromosome arm or acrocentric chromosome.

Interestingly, the NCDI of PositiveT-FC4 is significantly higher than corresponding values of PositiveT-FC3 in all analysed selected chromosomes (i.e., Chr20 in Figure 4A and Chr20q, Chr8, Chr8q, 13 and 7 in other selected COAD groups as reported in Supplementary Figure S2). Moreover, the NCDI values of PositiveT-FC3 in selected chromosomes are not significantly different from the NCDI of the entire repertoire of transcripts, with the exception of a slight increase in Chr20q (Figure 4A and Supplementary Figure S2). These data suggest that the relatively high density of up-regulated genes in selected chromosomes (Chr20, 8, 13 and 7) is mainly the consequence of the presence of BCNGs. Moreover, it should be noted that some chromosomes, such as Chr19 and Chr16, have a relatively high NCDI value, but do not show a high frequency of BCNGs. Therefore, a high density of cancer upregulated genes in disomic chromosomes cannot be considered one of the major predisposing factors for the development of the gain aberrations of those chromosomes during cancer progression. 

**Figure 5:**
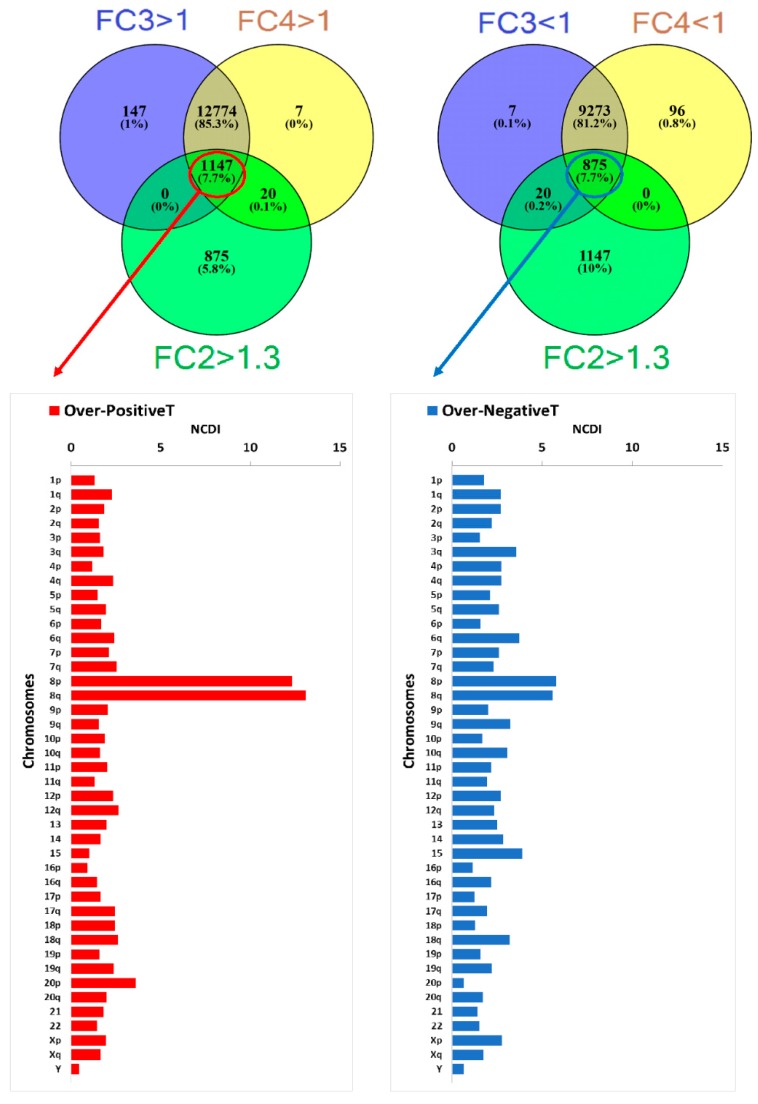
(Top) Venn Diagrams showing the number of transcripts expressing levels higher or lower than an established fold-change value. (Bottom) Normalized Chromosomal Distribution Index (NCDI) of Over-PositiveT (transcripts with FC2 > 1.3, FC3 > 1, FC4 > 1) or Over-NegativeT (transcripts with FC2 > 1.3, FC3 < 1, FC4 < 1) in all chromosomal arms and acrocentric chromosomes. Results have been obtained by analysing the Chr8 control COAD group, wChr8-gain COAD group and normal colonic mucosae.

**Figure 6:**
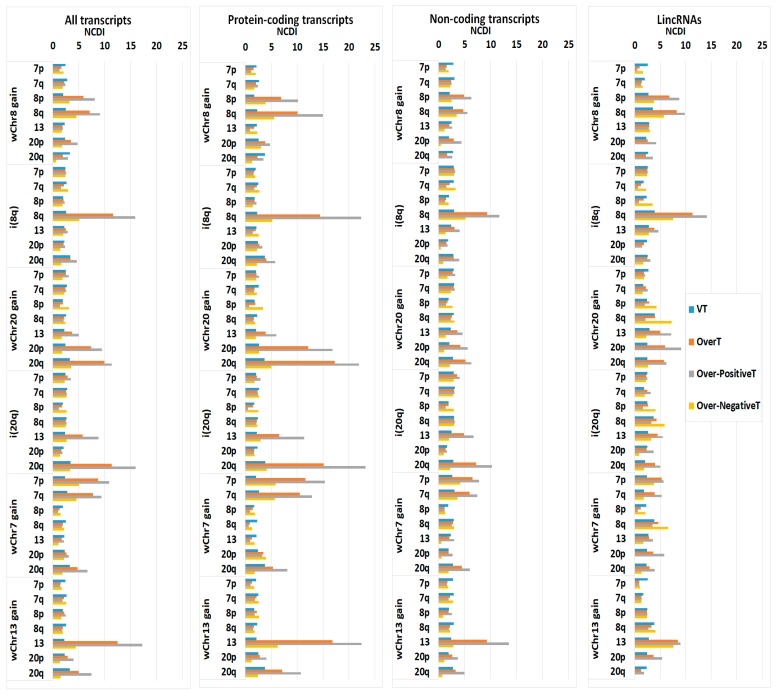
NCDI of different transcript classes (VT, OverT, Over-PositiveT, Over-NegativeT) in selected chromosomal arms or acrocentric chromosomes in different groups of CIN COAD samples. Calculation of NCDI values are reported for all transcript types or, separately, for “Protein-coding”, “Non-Coding” and “lincRNA”.

The number of transcripts bearing FC values higher than a predetermined threshold (FC2 > 1.3, FC3 > 1, FC4 > 1) are reported in the Venn diagrams in Figure 5 (wChr8-gain group) and in Supplementary Figure S3 (all examined COAD and CIN COAD subgroups). The transcripts contained in the region of overlapping of the three circles of the Venn diagram (corresponding to transcripts with FC2 > 1.3, FC3 > 1 and FC4 > 1) are called “Over-PositiveT”, because these transcripts are overexpressed when comparing the “wChr8-gain group”, with its corresponding “Chr8 Control group” (FC2 > 1.3), and show positive values of FC3 and FC4 (Figure 5). A high percentage of Over-PositiveT, as shown by the NCDI value, is localized in the gained chromosome characterizing the selected group (bottom of Figure 5, showing NCDI values for each of the chromosomal arms and acrocentric chromosome). Moreover, the NCDI value is lower for Over-NegativeT, defined as overexpressed transcripts with FC2 > 1.3, but showing FC3 < 1 and FC4 < 1 (Figure 5, Supplementary Figure S4 for all COAD samples, and Figure 6 for CIN COAD). The results of the hypergeometric test confirm an enrichment of OverT among PositiveT and/or their depletion among NegativeT (Table 1).

These data suggest a differential susceptibility of PositiveT and NegativeT to gene dosage effects. Bearing in mind that positive transcripts are up-regulated transcripts in comparison to normal tissue, and that negative ones are down-regulated transcripts, this phenomenon has been denominated the “positive caricature effect”, based on the definition of “caricature” as an exaggeration of tumour’s up-regulated gene expression features [19]. Of course, this is just a statistical observation, suggesting that the probability of gene-dosage effects in down-regulated genes is lower than in up-regulated ones.

Each selected COAD group’s three main transcript types were evaluated according to the annotation of GRCh38.p12 provided by the Ensembl BioMart tool (www.ensembl.org/biomart/): “Protein-coding”, “Non-Coding” and “lincRNA” (Long intergenic non-coding RNA). In the non-coding group, the TEC (To be Experimentally Confirmed) and NA (Not Assessed) entries were excluded. As shown in Figure 6, all three transcript types show an increase of the NCDI values of OverT and Over-PositiveT in the chromosome characterizing the “selected COAD group”, although the effect is more evident when analysing protein-coding transcripts. Moreover, Over-PositiveT NCDI values are significantly higher than Over-NegativeT ones in all transcript types.

**Figure 7:**
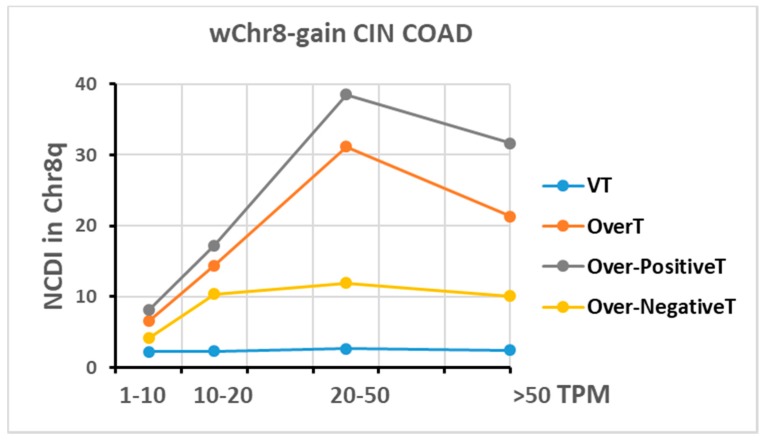
Transcripts are subdivided into different bins according to TPM values as indicated in the *x*-axis. The NCDI value in Chr8q is reported in the *y*-axis. Data obtained in the wChr8-gain CIN COAD group are reported.

In order to show that the difference between Over-PositiveT and Over-NegativeT is not simply dependent on the transcript level, transcripts were subdivided into different sets or bins according to their TPM (Transcripts Per Million) values, and NCDI values in Chr8q were calculated for each bin in the wChr8-gain COAD group (Figure 7). Although an increase of NCDI can be observed at higher TPM values, the NCDI value of Over-NegativeT is lower than that of overexpressed transcripts (OverT; FC2 > 1.3) or Over-PositiveT at all examined TPM bins. A similar result is observed for all the selected groups (Supplementary Figure S5).

### 2.5. Recurrent Gained Variant Enhancer Loci

Colon carcinogenesis is accompanied by locus-specific gains and losses of enhancer activity, called “Variant Enhancer Loci” (VELs) [28,29]. Cohen et al. [29] performed high-resolution H3K27ac ChIP-seq profiles in seven specimens of normal colonic epithelial crypts and 35 colorectal cancer samples. DNA sites in which the H3K27ac mark was more enriched in colorectal samples than in the normal crypts were termed “gained VELs”. Moreover, Cohen et al. [29] assigned VELs to their putative target genes and corresponding transcripts, by an experimentally validated computational method that predicts enhancer-gene interactions. Gained VELs that are present in 10 or more colorectal cancer samples were deemed as significantly recurrent, and transcripts linked to recurrent gained VELs (here denominated “gained VEL-T”) are up-regulated in primary colorectal cancer compared to normal tissue [29].

We report here that OverT are significantly enriched among gained VEL-T transcribed in selected chromosomal arms of selected COAD CIN groups (Table 1).

**Figure 8:**
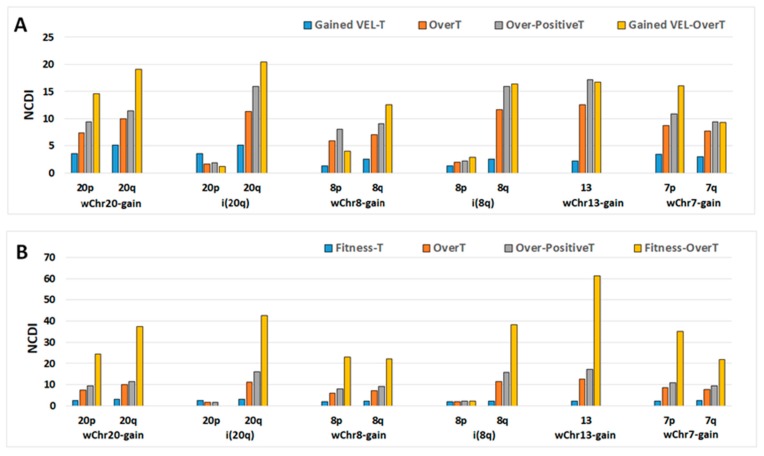
NCDI values of Gained Variant Enhancer Loci-Transcripts (VEL-T), OverT, Over-PositiveT, and Gained VEL-OverT (**A**), and Fitness-T, OverT, Over-PositiveT and Fitness-OverT (**B**) have been calculated in selected chromosomes of each Selected COAD group as indicated in the *x*-axis.

As shown in Figure 8, the NCDI value of Gained VEL-OverT is more elevated than that of OverT in chromosome arms 20p, 20q, 8q, 13, 7p and 7q, suggesting that epi-genomic changes associated with gained VEL may play a role in the susceptibility to the transcriptional dosage cis-effect. In several chromosomes (20p, 20q, 8q, 7p) the NCDI increases are higher than those observed in Over-PositiveT.

### 2.6. Cancer Fitness Genes

In order to identify genes that are required for cancer cell fitness, genome-scale CRISPR-Cas9 screens have been performed in cancer cell lines [30]. In particular, Behan et al. [30] identified genes required for cell growth or viability in colon cancer cell lines, whose transcripts are here abbreviated as “Fitness-T”. 

As shown in Table 1, OverT are significantly enriched among Fitness-T transcribed in selected gained chromosomal arms of selected COAD CIN groups. Moreover, NCDI values of Fitness-OverT are significantly higher than those of OverT and Over-PositiveT in selected chromosomal arms of each COAD group (Figure 8).

The Venn diagrams showing the overlapping of Over-positiveT, Fitness-OverT and Gained VEL-T, and the corresponding lists of associated genes, are reported in Supplementary Figure S6.

### 2.7. Recurrent Focal Amplifications

Sack et al. [11] have reported a list of genes included in recurrent focal amplification in colorectal adenocarcinomas. Although the focus of the present work is on arm-level gains or BCNGs, we wondered whether transcripts associated with focal amplifications (AmpT) are enriched among OverT genes identified in BCNG regions. As shown in Table 1, a trend towards an enrichment was observed in some chromosomes (20p, 8p, 13), but these results, with the exception of chr13, are not significant. Interestingly, some chromosome arms frequently involved in BCNGs do not show recurrent focal amplifications (8q, 7p), thus suggesting that fundamental differences distinguish the cancer driver mechanisms of broad and focal chromosomal aberrations. 

### 2.8. Ingenuity Pathway Analysis: Over-Positive T and Fitness-OverT

Ingenuity Pathway Analysis (IPA^®^) was performed using, as datasets, the lists of Over-PositiveT or Fitness-OverT obtained for each selected COAD group and the FC2 values. Each list of Over-PositiveT or Fitness-OverT contained only genes localized on a selected chromosome or chromosomal arm, as reported in Supplementary Table S1. In order to exploit data deriving from wChr-gain groups and isochromosomes, only genes located on the q arm of Chr20 and Chr8 were analysed. The IPA Core analysis identified several significant Canonical Pathways linked to cancer processes (Supplementary Table S1), such as pathways involved in proliferation and cell cycle control, cancer signalling, DNA repair, and amino acid metabolism. Proteins involved in the control of the cell cycle, such as E2F1 and RBL1 in Chr20, and CCNE2 and E2F5 in Chr8, are among the main determinants of such results. 

**Figure 9:**
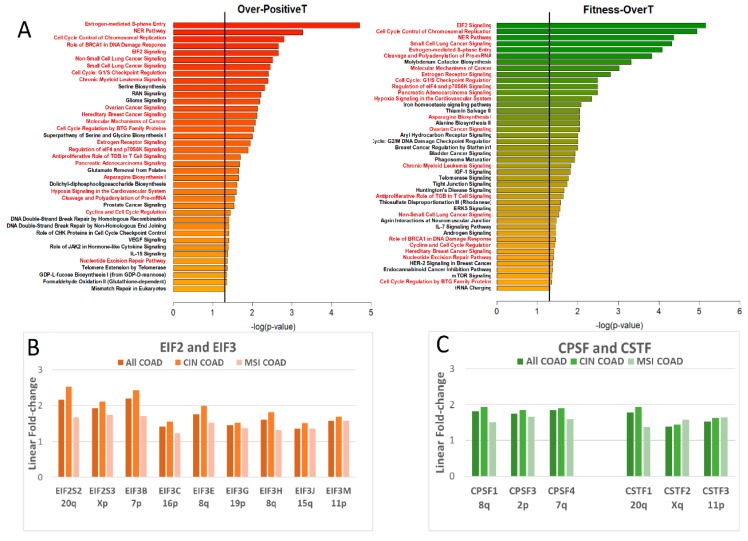
(**A**) The *p*-values of Canonical pathways significantly associated with Over-PositiveT (left) or Fitness- OverT (right) genes located in 20q, 8q, 13, 7. The vertical black line indicate a *p*-value equal to 0.05. Canonical pathways shared between Over-PositiveT and Fitness-OverT are written in red letters. (**B**) Expression level of some significantly up-regulated EIF2 and EIF3 genes in COAD samples. (**C**) Expression level of some significantly up-regulated CPSF and CSTF genes in COAD samples. In B and C, the expression level is expressed as linear fold-change in the comparison tumour vs. normal. Only genes significantly up-regulated (linear fold change >1.5 and False Discovery Rate (FDR) *p*-value < 0.05 in all COAD samples) are reported. Average values of 433 COAD samples, 223 CIN Coad and 60 MSI COAD are shown.

In order to explore the functional interactions between genes located in different chromosomes, we also prepared a combined list of Over-PositiveT or FitnessT-OverT genes located on Chr20q, 8q, 13 and 7. Results of IPA core analysis (Figure 9) revealed 39 and 44 significant canonical pathways for Over-positiveT and Fitness-OverT, respectively. Twenty-two canonical pathways are shared between the two transcript classes (in red letters in Figure 9). 

EIF2 signalling is one of the most significant pathways (top position for Fitness-OverT and 4th position for Over-PositiveT), with an activation Z-score > 2, indicating that the expression pattern of our dataset is consistent with the canonical pathway having more activity, according to the Ingenuity Knowledge Base^®^.

EIF2S2 (chr20q11.22), EIF3B (chr7p22.3), EIF3E (Chr8q23.1) and EIF3H (chr8 q24.11) are among the genes in our dataset that contribute to the identification of this EIF2 signalling pathway. In Figure 9B we report the expression levels of different EIF2 and EIF3 genes showing a significant increase in tumour versus normal tissue (linear fold change >1.5, FDR *p*-value < 0.05) in all COAD or in CIN and MSI COAD samples. Interestingly, EIF2S2, EIF2S3, EIF3B and EIF3E show the largest increase in expression in comparison to normal tissue. Moreover, the expression of several EIF2/3 genes in CIN tumours is higher than that of MSI ones.

“Cleavage and Polyadenylation of Pre-mRNA” is another significant pathway, in both Over-PositiveT and Fitness-OverT, which contains subunits of multi-protein complexes. One of these multi-subunit complexes is the cleavage and polyadenylation specificity factor (CPSF) playing a role in the 3′ processing of pre-mRNAs by the recognition of the AAUAAA signal and by the interaction with other complexes and enzymes involved in both RNA cleavage and poly(A) synthesis. CPSF includes the proteins CPSF1 (also known as CPSF160), CPSF2 (CPSF100), CPSF3 (CPSF73), CPSF4 (CPSF30), FIP1L1 and WDR33. Another multi-subunit complex belonging to the “Cleavage and Polyadenylation of Pre-mRNA pathway” is the “cleavage stimulation factor (CSTF)”, a trimer of CSTF1 (CstF50), CSTF2 (Cstf64) and CSTF3 (CstF77) [31,32,33]. In Figure 9C we report the expression levels of different genes of the CPSF and CSTF complexes, showing a significant increase in tumour versus normal tissue (linear fold change > 1.5, FDR *p*-value < 0.05) in all COAD or in CIN and MSI COAD samples. A significant difference between CIN and MSI COADs is observed for genes located in chromosomes undergoing frequent BCNGs, such as CPSF1 (chr8q24.3), CPSF4 (chr7q22.1) and CSTF1 (Chr20q13.2). Indeed, CPSF1, CPSF4 and CSTF1 are among genes in our dataset of Over-PositiveT and Fitness-OverT that contribute to the identification of the pathway “Cleavage and Polyadenylation of Pre-mRNA”.

## 3. Discussion

The effect of BCNGs on the level of transcripts encoded in the same chromosomal region (the gene-dosage transcriptional cis-effect) is well-known, and it has been reported in several published studies already quoted in the introduction see also references in [19]. The availability of SNP array and RNA-Seq data from a large number of TCGA colon adenocarcinoma samples characterized for genome instability allowed us to devise better control groups in order to identify changes in expression due to specific BCNGs. Indeed, the selective use of CIN samples allowed the generation of tumour control groups devoid of a specific BCNG, although displaying a similar range of other chromosomal aberrations. Our analysis shows that, in chromosomes selected for the presence of recurrent BCNGs, cancer up-regulated transcripts (PositiveT) are more likely to be affected by gene-dosage transcriptional effects than down-regulated ones (NegativeT) (Figure 5 and Figure 6). This phenomenon is also shown by the depletion of OverT among down-regulated transcripts (NegativeT) in BCNG-chromosomes (Table 1).

It is easy to conceive that changes in enhancer activity in down- or up-regulated genes can be determinants of the sensitivity to the gene-dosage transcriptional effect. The availability of genome-wide data on the markers of active chromatin in colorectal cancer [29] allowed us to test if the aberrant enhancer activity is associated with any sensitivity to BCNG-associated gene dosage effects. Indeed, a significant enrichment of BCNG-overexpressed genes among the so called “recurrent gained VEL” was observed in this work, suggesting that an active state of chromatin is one of the determinants of the gene-dosage transcriptional sensitivity. Moreover, a high density of recurrent gained VEL and super-enhancers have been observed in some chromosomes (such as chromosome 20 and 13) that undergo frequently to BCNGs in colon cancer. It is possible that the attainment of a saturation level in enhancer activity in cancer-related genes makes the increase in copy number the only efficient mechanism for a further increase of transcriptional activity.

It has been also reported that genes with a high expression level are more dosage sensitive than low-expressed genes [18]. Our data confirm this observation, but reveal that up-regulated transcripts (PositiveT) are more susceptible to gene-dosage effects than down-regulated ones (NegativeT), independently of transcript levels. 

Indeed, when transcripts are divided in different bins according to transcript level the difference in dosage sensitivity (measured as the chromosomal density of overexpressed transcripts in selected BCNG chromosomes) between PositiveT and NegativeT is maintained in all subsets (Figure 7). A similar result has been previously observed by using microarray technology for transcriptome analysis [19].

However, the increased BCNG-associated transcriptional activity does not necessarily translate in cancer growth advantageous features. Indeed, several downstream compensatory mechanisms are present, or the increased transcriptional activity is just a passenger feature that does not affect the functional pathways relevant for cancer progression. In agreement with a functional role in cancer progression, we have previously shown a significant enrichment of cancer-related genes among overexpressed and up-regulated genes [19]. In the present study we show that genes required for cell growth or viability in colon cancer cell lines, identified by genome-scale CRISPR-Cas9 screens [30], and called fitness genes, are significantly enriched among BCNG-overexpressed genes. Indeed, the overexpression of specific “cancer fitness” genes might represent an important step in the mechanism underlying the role of recurrent BCNGs in cancer evolution. Hart et al. [20] report some examples of fitness genes that have been found to be amplified across several cancer tissues and cell lines.

Our data on the relationship between colon cancer fitness genes and recurrent BCNGs are in agreement with recent data obtained by a gain-of-function screening with a modular ORF expression system [11]. This screening effort is, somewhat, complementary to the inactivating CRISPR-Cas9 screening of Behan et al. [30]. Sack et al. [11] define experimentally overexpressed genes that significantly enhance cancer proliferation as GO genes. They show that taking in consideration GO genes significantly improved the predictions of the frequency of deletions and amplifications of chromosomal arms and whole chromosomes within a pan-cancer tumour set.

Recognition of overexpressed cancer fitness genes in selected chromosomes undergoing recurrent BCNGs, such as Chr20, 8, 13 and 7, allowed a combined pathway analysis of this gene list and the identification of several significant canonical pathways linked to cancer progression. Indeed, intra-chromosomal and inter-chromosomal cooperation of multiple genes can explain the recurrence of BCNGs and their frequent association in colon cancer. As an example, we have here reported the highly significant EIF2 signalling pathway, identified by the contribution of genes EIF2S2 (chr20q11.22), EIF3B (chr7p22.3), EIF3E (Chr8q23.1) and EIF3H (chr8 q24.11). These genes encode eukaryotic initiation factors (eIFs), which are protein complexes involved in the initiation phase of translation. In particular, the EIF2S2 gene encodes one of the subunits of eIF2 (eIF2β). EIF2 is composed of three subunits (α, β, γ), forms a ternary complex with GTP and the initiator tRNA, and binds to a 40S ribosomal subunit. This eIF3 is formed of 13 subunits (a-m) and participates in the assembly of the 40S ribosomal subunit on mRNA, thus forming a functional pre-initiation complex. It has been reported that eIF3 binds to the 5’ untranslated region of a specific set of mRNAs involved in cell growth control [34], and that the increased expression of EIF3H gene potentiates colorectal cancer growth and invasiveness [35]. Interestingly, another eukaryotic translation initiation factor, eIF6, is included in the list of Over-PositiveT and Fitness-OverT genes located in Chr20 (Supplementary Figure S6). This result is in agreement with our previous data by microarray transcriptomics [19] and Gandin et al. [36] have already reported that this factor is rate-limiting in translation, growth and transformation.

Another significant pathway identified through the analysis of Over-PositiveT and Fitness-OverT is the “Cleavage and Polyadenylation of Pre-mRNA”, that contains multiple subunits of CPSF and CSTF complexes. In particular, our analysis identified CPSF1, CPSF4 and CSTF1 among Fitness-OverT genes. Interestingly, Yang et al. [37] have recently reported that a knockdown of CSPF4 in human colorectal cancer cell lines inhibited cell proliferation, migration, invasion and stemness maintenance. Moreover, ectopic overexpression of CPSF4 enhanced tumour growth in mouse models with colon cancer xenografts [37]. Behan et al. [30] classified CPSF1, CPSF4 and CSTF1 among context-specific fitness genes. In other words, those genes are required for cell fitness in specific molecular or histological contexts, in contrast with “core fitness” genes, that play an essential role in all cell types. 

This observation can be important in the selection of drug targets, because the inhibition of context-specific fitness genes has a reduced likelihood of inducing toxic effects in healthy tissues.

In conclusion, our data suggest the hypothesis that an overexpression of specific cancer fitness genes might play a significant role in the functional selection of gained chromosomal arms during cancer evolution. Therefore, Fitness-OverT, as defined in the present paper, might represent candidate targets for a silencing transcriptional therapy in tumours bearing specific BCNGs. These genes have already been shown to be functionally involved in cancer proliferation by the CRISPR-Cas9 inactivation, but the anticancer effects of their partial and combined downregulation remain to be investigated.

## 4. Materials and methods

### 4.1. RNA-Seq Data Retrieval from GDC Data Portal

The Colon adenocarcinoma (COAD) samples were downloaded from *The Genomic Data Commons (GDC) Data Portal* (https://portal.gdc.cancer.gov) by selecting the RNA-Seq counts data from The Cancer Genome Atlas (TCGA), PanCancer program) [38,39]. We selected 480 COAD samples and 41 mucosal normal samples (*n* = 521). The data were filtered by selecting only primary tumour (-01A) tissue type and removing the other types (-01B, -01C, -02A, -06A), thus providing 461 tumour samples.

### 4.2. Single-Nucleotide Polymorphism (SNP) Arrays Data Retrieval from Cbioportal

The cytogenetic results (Affymetrix SNP 6.0 arrays) of 439 samples were downloaded from *cBioPortal for cancer genomics* (https://www.cbioportal.org) [23,24] but only 433 samples matched to TCGA-COAD RNA-Seq data were selected for further analysis. 

### 4.3. Statistical Analysis of Differential Expression by RNA-Seq Data

All count data were normalized with the “trimmed mean of M-values” method introduced by Robinson and Oshlack [40]. Differential expression of transcripts was analysed by the R packages edgeR and compcodeR [26,27,41]. The packages perform a statistical method for multiple experiments obtaining the log-fold changes between two conditions. The *p*-values were adjusted for multiple comparisons by using the Benjamini-Hochberg correction [42]. The log-fold changes were then transformed into linear fold-changes, and fold changes < 1 have been reported as the negative of the reciprocal (e.g., a fold change of ½ is reported as −2). 

### 4.4. Normalized Chromosomal Distribution Index (NCDI)

The number of transcripts belonging to a specific **“transcript class”** (for instance PositiveT) and transcribed in a specific chromosome arm or acrocentric chromosome is also reported as a percentage of the total number of transcripts belonging to that class in the whole genome, thus obtaining the chromosomal distribution index (CDI) of that transcript class among 42 different chromosomal regions. As previously described in Condorelli et al. [19], CDI has been normalised for the total number of transcripts belonging to a chromosomal region (NCDI):

$$\mathrm{NCDI}\;\mathrm{of}\;\mathrm{chromosomal}\;\mathrm{region}\;n\;=\frac{x_{n}}{X_{n}}*\frac{1}{\sum_{i=1}^{T}\frac{x_{i}}{X_{i}}}*100$$

where *x_n_* is the number of the transcript belonging to a specific transcript class in the *n*th chromosomal region, $X_{n}$ is the total number of transcripts in the *n*th chromosomal region, *T* is the total number of chromosomal regions subdividing the entire genome (42 in this report). The ratio between *x_n_* and $X_{n}$ represents the chromosomal density of the transcript class.

### 4.5. Bioinformatics and Statistical Tools

The data management (i.e., matching SNP-arrays with RNA-Seq, tumour groups) was performed by using SQL queries. The Venn-diagrams were created by using the Venny 2.0 online tool [43]. Pathway analysis was performed by the IPA software (QIAGEN Inc., https://www.qiagenbioinformatics.com/products/ingenuitypathway-analysis). The hypergeometric tests were performed by the online hypergeometric *p*-value calculator of the Graeber Lab (systems.crump.ucla.edu/hypergeometric/).

## Figures and Tables

**Table 1:** Fold-enrichment of OverT among different classes of transcripts evaluated by hypergeometric test.

	Chr20p	Chr20q	Chr8p	Chr8q	Chr13	Chr7p	Chr7q
Fold-enrichment of OverT among PositiveT (Hypergeometric *p*-value)	1.11 (0.00040)	1.00 (0.49343)	1.16 (0.00109)	1.08 (0.02179)	1.12 (2.93 × 10^−7^)	1.10 (0.02120)	1.05 (0.14272)
Fold-depletion of OverT among NegativeT (Hypergeometric *p*-value)	−2.69 (4.55 × 10^−11^)	−1.6 (3.30 × 10^−7^)	−1.45 (0.00109)	−1.19 (0.01940)	−1.8 (5.94 × 10^−11^)	−1.38 (0.00331)	−1.3 (0.00653)
Fold-enrichment of OverT among Gained VEL-T (Hypergeometric *p*-value)	2.14 (2.23 × 10^−6^)	1.45 (0.00021)	1.99 (0.03708)	3.29 (1.87 × 10^−13^)	2.25 (2.14 × 10^−7^)	2.33 (1.85 × 10^−6^)	1.76 (0.00151)
Fold-enrichment of OverT among Fitness-T (Hypergeometric *p*-value)	2.43 (5.03 × 10^−7^)	2.22 (5.55 × 10^−15^)	4.41 (2.31 × 10^−13^)	3.14 (1.5 × 10^−12^)	3.58 (5.81 × 10^−26^)	2.61 (4.88 × 10^−6^)	1.65 (0.00929)
Fold-enrichment of OverT among AmpT (Hypergeometric *p*-value)	9.75 (6.56 × 10^−6^)	1.15 (0.109)	3.73 (0.250)	NC ^1^	2.32 (0.002)	NC ^1^	1.07 (0.523)
^1^ not computable for the absence of AmpT.							

## Supplementary Materials

Supplementary Materials can be found at https://www.mdpi.com/1422-0067/20/18/4652/s1.

## Abbreviations

COAD	COlon ADenocarcinoma
CIN	Chromosomal INstability
MSI	MicroSatellite Instability
BCNGs	Broad Copy Number Gains
VT	Variable Transcripts
PositiveT	Positive Transcripts
NegativeT	Negative Transcripts
OverT	Overexpressed Transcripts
FC	Fold-Change
NCDI	Normalized Chromosomal Distribution Index
Over-PositiveT	Overexpressed Positive Transcripts
Over-NegativeT	Overexpressed Negative transcripts
VELs	Variant Enhancer Loci
Gained VEL-T	Transcripts linked to recurrent gained VELs
