# Supplementary material for: Chromosomal Density of Cancer Up-Regulated Genes, Aberrant Enhancer Activity and Cancer Fitness Genes Are Associated with Transcriptional Cis-Effects of Broad Copy Number Gains in Colorectal Cancer
Source: Int J Mol Sci. 2019 Sep 19;20(18):4652. doi: 10.3390/ijms20184652 (PMC6770609; doi:10.3390/ijms20184652)
Supplement: Supplementary file 1 [file ijms-20-04652-s001.zip › SupplementaryTable 1.docx]

|  | Over-PositiveT in selected CIN COAD group | | | | | |
| --- | --- | --- | --- | --- | --- | --- |
|  | wChr20-gain | | | i(20q) | | |
| chromosome | 20q | | | 20q | | |
| INGENUITY PATHWAYS ANALYSIS | Canonical Pathways | p-value | Molecules | Canonical Pathways | p-value | Molecules |
|  | Estrogen-mediated S-phase Entry | 8.71E-03 | E2F1,RBL1 | Estrogen-mediated  S-phase Entry | 1.07E-02 | E2F1,RBL1 |
|  | Non-Small Cell Lung Cancer Signaling | 1.10E-02 | STK4,E2F1,PLCG1 | Non-Small Cell Lung Cancer Signaling | 1.51E-02 | STK4,E2F1,PLCG1 |
|  | VEGF Signaling | 2.24E-02 | BCL2L1,PLCG1,EIF2S2 | VEGF Signaling | 3.02E-02 | BCL2L1,PLCG1,EIF2S2 |
|  | Chronic Myeloid Leukemia Signaling | 2.34E-02 | BCL2L1,E2F1,RBL1 | Chronic Myeloid Leukemia Signaling | 3.16E-02 | BCL2L1,E2F1,RBL1 |
|  | Glioma Signaling | 2.82E-02 | E2F1,PLCG1,RBL1 | Glioma Signaling | 3.89E-02 | E2F1,PLCG1,RBL1 |
|  |  |  |  | Pancreatic Adenocarcinoma Signaling | 3.89E-02 | BCL2L1,E2F1,MMP9 |
|  | wChr8-gain | | | i(8q) | | |
| chromosome | 8q | | | 8q | | |
| INGENUITY PATHWAYS ANALYSIS | Canonical Pathways | p-value | Molecules | Canonical Pathways | p-value | Molecules |
|  | EIF2 Signaling | 7.41E-05 | PABPC1,RPL8,EIF3H,RPS20,RPL30,EIF3E,RPL7 | EIF2 Signaling | 3.47E-05 | MYC,PABPC1,RPL8,EIF3H,RPS20,RPL30,AGO2,EIF3E,RPL7 |
|  | DNA Double-Strand Break Repair by Non-Homologous End Joining | 1.74E-03 | PRKDC,NBN | Estrogen-mediated S-phase Entry | 7.76E-04 | MYC,CCNE2,E2F5 |
|  | Endocannabinoid Cancer Inhibition Pathway | 5.50E-03 | SMPD5,PTK2,CCNE2,NSMAF | DNA Double-Strand Break Repair by Non-Homologous End Joining | 4.17E-03 | PRKDC,NBN |
|  | Antiproliferative Role of TOB in T Cell Signaling | 5.89E-03 | PABPC1,CCNE2 | Endocannabinoid Cancer Inhibition Pathway | 5.13E-03 | MYC,SMPD5,PTK2,CCNE2,NSMAF |
|  | Estrogen-mediated S-phase Entry | 5.89E-03 | CCNE2,E2F5 | Regulation of eIF4 and p70S6K Signaling | 6.17E-03 | PABPC1,EIF3H,RPS20,AGO2,EIF3E |
|  | Regulation of eIF4 and p70S6K Signaling | 6.31E-03 | PABPC1,EIF3H,RPS20,EIF3E | Polyamine Regulation in Colon Cancer | 1.02E-02 | MYC,AZIN1 |
|  | Molecular Mechanisms of Cancer | 8.32E-03 | PTK2,PRKDC,CCNE2,FZD6,E2F5,NBN | Cell Cycle: G1/S Checkpoint Regulation | 1.15E-02 | MYC,CCNE2,E2F5 |
|  | Retinoate Biosynthesis I | 1.00E-02 | RDH10,SDR16C5 | Antiproliferative Role of TOB in T Cell Signaling | 1.41E-02 | PABPC1,CCNE2 |
|  | Cell Cycle Regulation by BTG Family Proteins | 1.17E-02 | CCNE2,E2F5 | Estrogen Receptor Signaling | 1.45E-02 | PRKDC,MED30,POLR2K,TAF2 |
|  | HIF1alpha Signaling | 1.86E-02 | MAPK15,COPS5,ELOC | Molecular Mechanisms of Cancer | 2.09E-02 | MYC,PTK2,PRKDC,CCNE2,E2F5,FZD6,NBN |
|  | Role of CHK Proteins in Cell Cycle Checkpoint Control | 2.69E-02 | E2F5,NBN | Small Cell Lung Cancer Signaling | 2.34E-02 | PTK2,MYC,CCNE2 |
|  | Sphingomyelin Metabolism | 3.47E-02 | SMPD5 | Retinoate Biosynthesis I | 2.34E-02 | RDH10,SDR16C5 |
|  | Cell Cycle: G1/S Checkpoint Regulation | 3.63E-02 | CCNE2,E2F5 | Cell Cycle Regulation by BTG Family Proteins | 2.75E-02 | CCNE2,E2F5 |
|  | Hypoxia Signaling in the Cardiovascular System | 4.27E-02 | COPS5,UBE2V2 | Ceramide Signaling | 3.39E-02 | SMPD5,NSMAF,TNFRSF11B |
|  | Role of BRCA1 in DNA Damage Response | 4.90E-02 | E2F5,NBN | Assembly of RNA Polymerase II Complex | 4.79E-02 | POLR2K,TAF2 |
|  | wChr13-gain | | | wChr7-gain | | |
| chromosome | 13 | | | 7 | | |
| INGENUITY PATHWAYS ANALYSIS | Canonical Pathways | p-value | Molecules | Canonical Pathways | p-value | Molecules |
|  | Non-Small Cell Lung Cancer Signaling | 1.62E-03 | RB1,RAP2A,TFDP1,IRS2 | Cell Cycle Control of Chromosomal Replication | 3.09E-04 | RPA3,CDK5,ORC5,MCM7 |
|  | Ovarian Cancer Signaling | 2.04E-03 | RB1,RAP2A,TFDP1,BRCA2,IRS2 | NER Pathway | 3.02E-03 | RPA3,COPS6,POLR2J,POLD2 |
|  | Hereditary Breast Cancer Signaling | 2.09E-03 | RB1,RAP2A,BRCA2,IRS2,RFC3 | Role of JAK2 in Hormone-like Cytokine Signaling | 1.62E-02 | EPO,SH2B2 |
|  | Prostate Cancer Signaling | 3.24E-03 | RB1,RAP2A,TFDP1,IRS2 | Nucleotide Excision Repair Pathway | 1.74E-02 | RPA3,POLR2J |
|  | RAN Signaling | 4.47E-03 | KPNA3,IPO5 | RhoA Signaling | 3.47E-02 | ARPC1B,EPHA1,LIMK1 |
|  | Chronic Myeloid Leukemia Signaling | 4.57E-03 | RB1,RAP2A,TFDP1,IRS2 | Amyloid Processing | 3.47E-02 | CDK5,PRKAR1B |
|  | Glioma Signaling | 5.89E-03 | RB1,RAP2A,TFDP1,IRS2 | Semaphorin Signaling in Neurons | 3.72E-02 | CDK5,LIMK1 |
|  | Pancreatic Adenocarcinoma Signaling | 5.89E-03 | RB1,TFDP1,BRCA2,IRS2 | Sperm Motility | 3.80E-02 | ZP3,ZAN,PRKAR1B |
|  | Melanoma Signaling | 6.17E-03 | RB1,RAP2A,IRS2 | Iron homeostasis signaling pathway | 4.47E-02 | EPO,ATP6V1F,TFR2 |
|  | Estrogen Receptor Signaling | 8.32E-03 | RAP2A,CDK8,GTF2F2,MED4 | SPINK1 Pancreatic Cancer Pathway | 4.68E-02 | CPA5,PRSS1 |
|  | HMGB1 Signaling | 9.77E-03 | HMGB1,RAP2A,IL17D,IRS2 | Ephrin A Signaling | 5.01E-02 | EPHA1,LIMK1 |
|  | Estrogen-mediated S-phase Entry | 1.02E-02 | RB1,TFDP1 |  |  |  |
|  | Role of BRCA1 in DNA Damage Response | 1.20E-02 | RB1,BRCA2,RFC3 |  |  |  |
|  | Neurotrophin/TRK Signaling | 1.35E-02 | RAP2A,SPRY2,IRS2 |  |  |  |
|  | Small Cell Lung Cancer Signaling | 1.48E-02 | RB1,TFDP1,IRS2 |  |  |  |
|  | Bladder Cancer Signaling | 1.82E-02 | RB1,RAP2A,TFDP1 |  |  |  |
|  | Role of p14/p19ARF in Tumor Suppression | 2.88E-02 | RB1,IRS2 |  |  |  |
|  | Telomerase Signaling | 3.39E-02 | RB1,RAP2A,IRS2 |  |  |  |
|  | Gap Junction Signaling | 3.47E-02 | RAP2A,GJB6,TUBA3C/TUBA3D,IRS2 |  |  |  |
|  | Rac Signaling | 3.72E-02 | MCF2L,RAP2A,IRS2 |  |  |  |
|  | 14-3-3-mediated Signaling | 4.90E-02 | RAP2A,TUBA3C/TUBA3D,IRS2 |  |  |  |

|  | Fitness-OverT in selected CIN COAD groups | | | | | | | | |
| --- | --- | --- | --- | --- | --- | --- | --- | --- | --- |
|  | wChr20-gain | | | | i(20q) | | | | |
| chromosome | 20q | | | | 20q | | | | |
| INGENUITY PATHWAYS ANALYSIS | Canonical Pathway | | p-value | Molecules | Canonical Pathways | | p-value | Molecules | |
|  | Molybdenum Cofactor Biosynthesis | | 2.88E-05 | MOCS3,NFS1 | Molybdenum Cofactor Biosynthesis | | 2.75E-05 | MOCS3,NFS1 | |
|  | Small Cell Lung Cancer Signaling | | 1.12E-02 | BCL2L1,E2F1 | Small Cell Lung Cancer Signaling | | 1.07E-02 | BCL2L1,E2F1 | |
|  | Hypoxia Signaling in the Cardiovascular System | | 1.17E-02 | UBE2C,UBE2V1 | Hypoxia Signaling in the Cardiovascular System | | 1.12E-02 | UBE2C,UBE2V1 | |
|  | p53 Signaling | | 2.00E-02 | BCL2L1,E2F1 | p53 Signaling | | 1.91E-02 | BCL2L1,E2F1 | |
|  | VEGF Signaling | | 2.09E-02 | BCL2L1,EIF2S2 | VEGF Signaling | | 2.04E-02 | BCL2L1,EIF2S2 | |
|  | Chronic Myeloid Leukemia Signaling | | 2.24E-02 | BCL2L1,E2F1 | Protein Ubiquitination Pathway | | 2.14E-02 | PSMA7,UBE2C,UBE2V1 | |
|  | Protein Ubiquitination Pathway | | 2.24E-02 | PSMA7,UBE2C,UBE2V1 | Chronic Myeloid Leukemia Signaling | | 2.14E-02 | BCL2L1,E2F1 | |
|  | Pancreatic Adenocarcinoma Signaling | | 2.45E-02 | BCL2L1,E2F1 | Pancreatic Adenocarcinoma Signaling | | 2.34E-02 | BCL2L1,E2F1 | |
|  | Regulation of eIF4 and p70S6K Signaling | | 4.79E-02 | EIF2S2,RPS21 | Regulation of eIF4 and p70S6K Signaling | | 4.57E-02 | EIF2S2,RPS21 | |
|  | wChr8-gain | | | | i(8q) | | | | |
| chromosome | 8q | | | | 8q | | | | |
| INGENUITY PATHWAYS ANALYSIS | Canonical Pathway | p-value | | Molecules | Canonical Pathways | p-value | | Molecules | |
|  | EIF2 Signaling | 1.82E-06 | | EIF3E,PABPC1,RPL30,RPL7,RPL8,RPS20 | EIF2 Signaling | 6.92E-07 | | | EIF3E,MYC,PABPC1,RPL30,RPL7,RPL8,RPS20 |
|  | Antiproliferative Role of TOB in T Cell Signaling | 8.51E-04 | | CCNE2,PABPC1 | Small Cell Lung Cancer Signaling | 5.75E-04 | | | CCNE2,MYC,PTK2 |
|  | Regulation of eIF4 and p70S6K Signaling | 2.29E-03 | | EIF3E,PABPC1,RPS20 | Antiproliferative Role of TOB in T Cell Signaling | 1.58E-03 | | | CCNE2,PABPC1 |
|  | Small Cell Lung Cancer Signaling | 6.46E-03 | | CCNE2,PTK2 | Estrogen-mediated S-phase Entry | 1.58E-03 | | | CCNE2,MYC |
|  | IL-7 Signaling Pathway | 7.76E-03 | | IL7,PTK2 | NER Pathway | 1.62E-03 | | | COPS5,POLR2K,TCEA1 |
|  | NER Pathway | 1.29E-02 | | COPS5,TCEA1 | Estrogen Receptor Signaling | 3.63E-03 | | | MED30,POLR2K,TAF2 |
|  | Endocannabinoid Cancer Inhibition Pathway | 2.40E-02 | | CCNE2,PTK2 | Endocannabinoid Cancer Inhibition Pathway | 4.17E-03 | | | CCNE2,MYC,PTK2 |
|  | mTOR Signaling | 4.79E-02 | | EIF3E,RPS20 | Regulation of eIF4 and p70S6K Signaling | 5.50E-03 | | | EIF3E,PABPC1,RPS20 |
|  |  |  | |  | Assembly of RNA Polymerase II Complex | 5.75E-03 | | | POLR2K,TAF2 |
|  |  |  | |  | ERK/MAPK Signaling | 9.55E-03 | | | MYC,PTK2,YWHAZ |
|  |  |  | |  | Myc Mediated Apoptosis Signaling | 9.77E-03 | | | MYC,YWHAZ |
|  |  |  | |  | Cell Cycle: G1/S Checkpoint Regulation | 1.00E-02 | | | CCNE2,MYC |
|  |  |  | |  | ERK5 Signaling | 1.15E-02 | | | MYC,YWHAZ |
|  |  |  | |  | Hypoxia Signaling in the Cardiovascular System | 1.23E-02 | | | COPS5,UBE2W |
|  |  |  | |  | IL-7 Signaling Pathway | 1.38E-02 | | | MYC,PTK2 |
|  |  |  | |  | HIPPO signaling | 1.58E-02 | | | STK3,YWHAZ |
|  |  |  | |  | IGF-1 Signaling | 2.34E-02 | | | PTK2,YWHAZ |
|  |  |  | |  | Telomerase Signaling | 2.45E-02 | | | MYC,TERF1 |
|  |  |  | |  | Androgen Signaling | 3.80E-02 | | | POLR2K,TAF2 |
|  |  |  | |  | Iron homeostasis signaling pathway | 3.80E-02 | | | ATP6V1C1,ATP6V1H |
|  |  |  | |  | Aryl Hydrocarbon Receptor Signaling | 4.17E-02 | | | CCNE2,MYC |
|  |  |  | |  | Phagosome Maturation | 4.47E-02 | | | ATP6V1C1,ATP6V1H |
|  | wChr13-gain | | | | wChr7-gain | | | | |
| chromosome | 13 | | | | 7 | | | | |
| INGENUITY PATHWAYS ANALYSIS | Canonical Pathway | p-value | | Molecules | Canonical Pathways | p-value | | | Molecules |
|  | Estrogen Receptor Signaling | 4.57E-03 | | CDK8,GTF2F2,MED4 | Cell Cycle Control of Chromosomal Replication | 4.57E-06 | | | CDK5,MCM7,ORC5,RPA3 |
|  | Role of BRCA1 in DNA Damage Response | 1.62E-02 | | BRCA2,RFC3 | NER Pathway | 1.12E-03 | | | COPS6,POLD2,RPA3 |
|  | Pancreatic Adenocarcinoma Signaling | 2.95E-02 | | BRCA2,TFDP1 | Huntington's Disease Signaling | 1.26E-03 | | | CDK5,EGFR,GNB2,YKT6 |
|  | Adipogenesis pathway | 4.27E-02 | | KLF5,SAP18 | G Beta Gamma Signaling | 1.82E-03 | | | EGFR,GNB2,PRKAG2 |
|  | Hereditary Breast Cancer Signaling | 4.57E-02 | | BRCA2,RFC3 | Tight Junction Signaling | 4.47E-03 | | | CPSF4,PRKAG2,YKT6 |
|  | Ovarian Cancer Signaling | 4.57E-02 | | BRCA2,TFDP1 | Amyloid Processing | 4.57E-03 | | | CDK5,PRKAG2 |
|  |  |  | |  | Colorectal Cancer Metastasis Signaling | 1.38E-02 | | | EGFR,GNB2,PRKAG2 |
|  |  |  | |  | IL-1 Signaling | 1.41E-02 | | | GNB2,PRKAG2 |
|  |  |  | |  | alpha-Adrenergic Signaling | 1.51E-02 | | | GNB2,PRKAG2 |
|  |  |  | |  | Neuregulin Signaling | 1.55E-02 | | | CDK5,EGFR |
|  |  |  | |  | G-alpha-s Signaling | 1.91E-02 | | | GNB2,PRKAG2 |
|  |  |  | |  | CDK5 Signaling | 1.95E-02 | | | CDK5,PRKAG2 |
|  |  |  | |  | Synaptogenesis Signaling Pathway | 2.40E-02 | | | CDK5,PRKAG2,YKT6 |
|  |  |  | |  | G-alphai Signaling | 2.57E-02 | | | GNB2,PRKAG2 |
|  |  |  | |  | P2Y Purigenic Receptor Signaling Pathway | 2.69E-02 | | | GNB2,PRKAG2 |
|  |  |  | |  | Androgen Signaling | 3.02E-02 | | | GNB2,PRKAG2 |
|  |  |  | |  | Iron homeostasis signaling pathway | 3.02E-02 | | | ATP6V1F,EGFR |
|  |  |  | |  | Ovarian Cancer Signaling | 3.16E-02 | | | EGFR,PRKAG2 |
|  |  |  | |  | Cardiac Beta-adrenergic Signaling | 3.16E-02 | | | GNB2,PRKAG2 |
|  |  |  | |  | Relaxin Signaling | 3.55E-02 | | | GNB2,PRKAG2 |
|  |  |  | |  | Phagosome Maturation | 3.55E-02 | | | ATP6V1F,YKT6 |
|  |  |  | |  | Dopamine-DARPP32 Feedback in cAMP Signaling | 4.17E-02 | | | CDK5,PRKAG2 |
|  |  |  | |  | GNRH Signaling | 4.68E-02 | | | EGFR,PRKAG2 |
