# Supplementary material for: Chromosomal Density of Cancer Up-Regulated Genes, Aberrant Enhancer Activity and Cancer Fitness Genes Are Associated with Transcriptional Cis-Effects of Broad Copy Number Gains in Colorectal Cancer
Source: Int J Mol Sci. 2019 Sep 19;20(18):4652. doi: 10.3390/ijms20184652 (PMC6770609; doi:10.3390/ijms20184652)

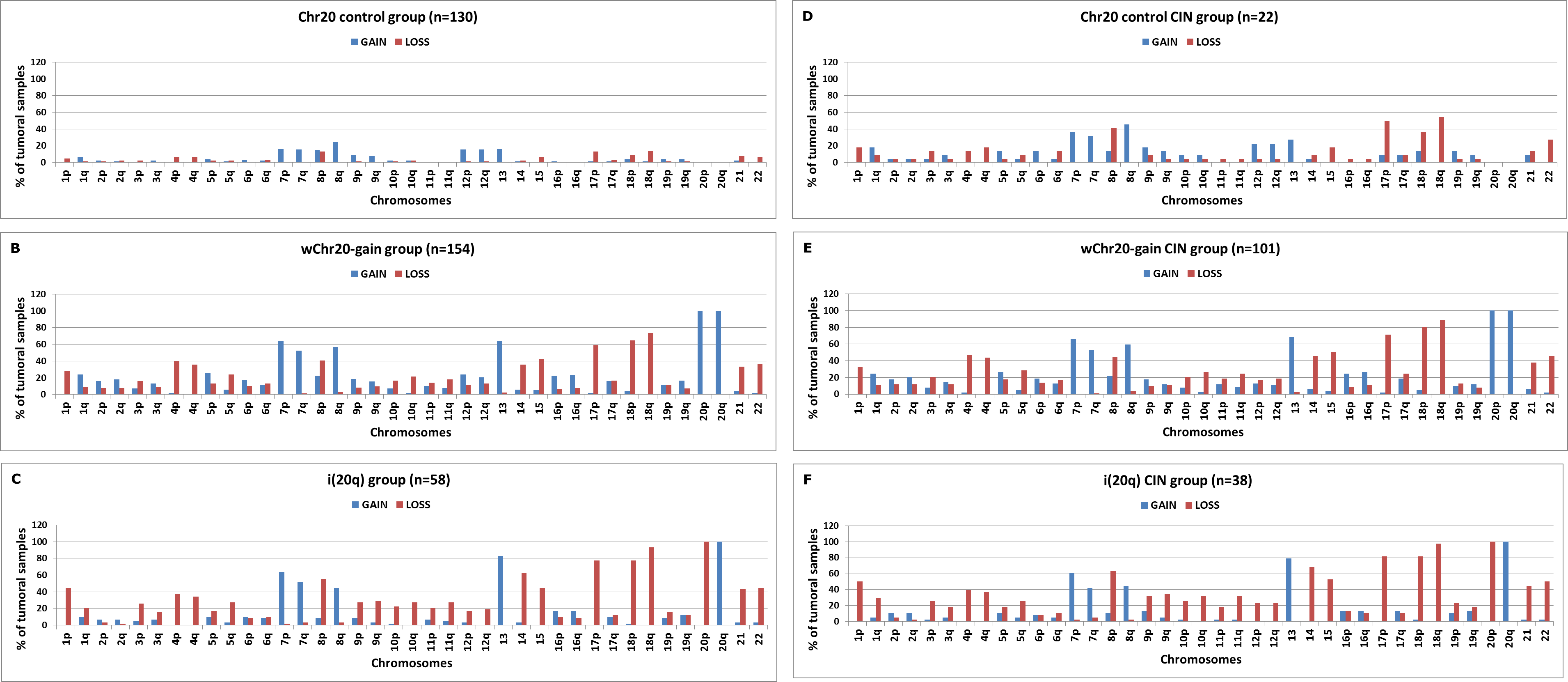
Chromosome 20

**A**

Chromosome 7


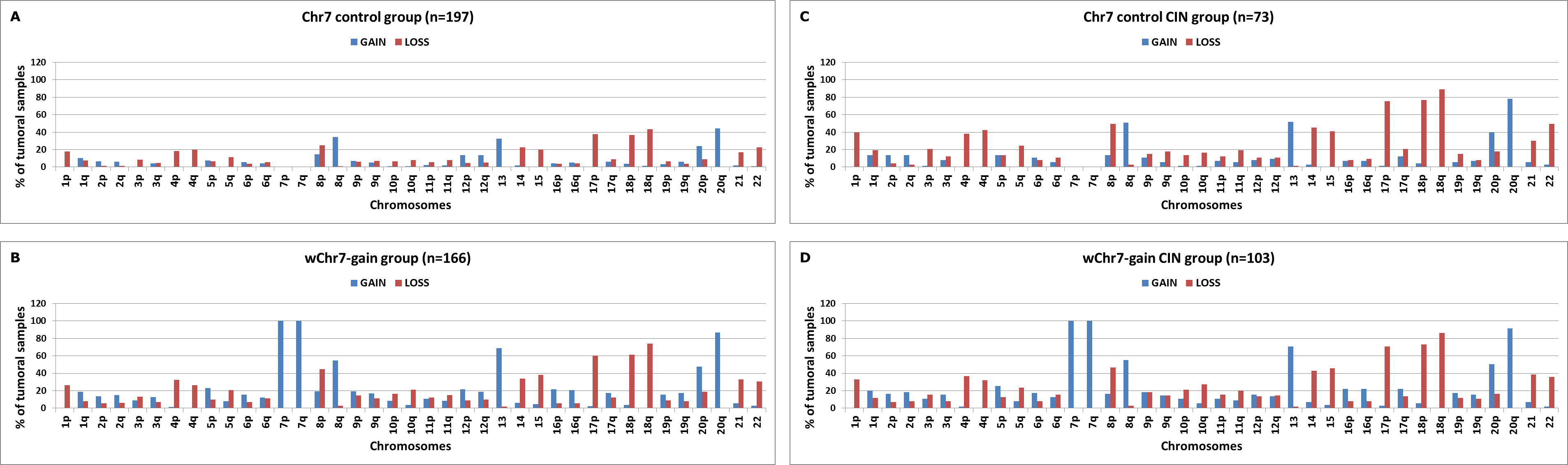


Chromosome 13


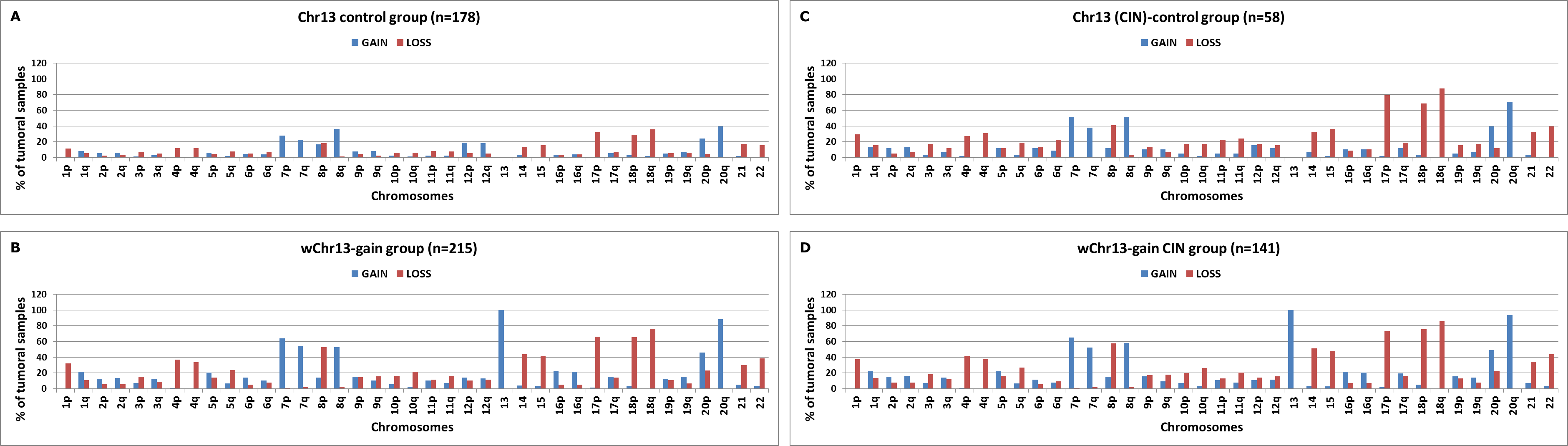


**Correlation plots of frequencies of arm-level gains and losses between selected CIN COAD groups and control CIN COAD groups.**


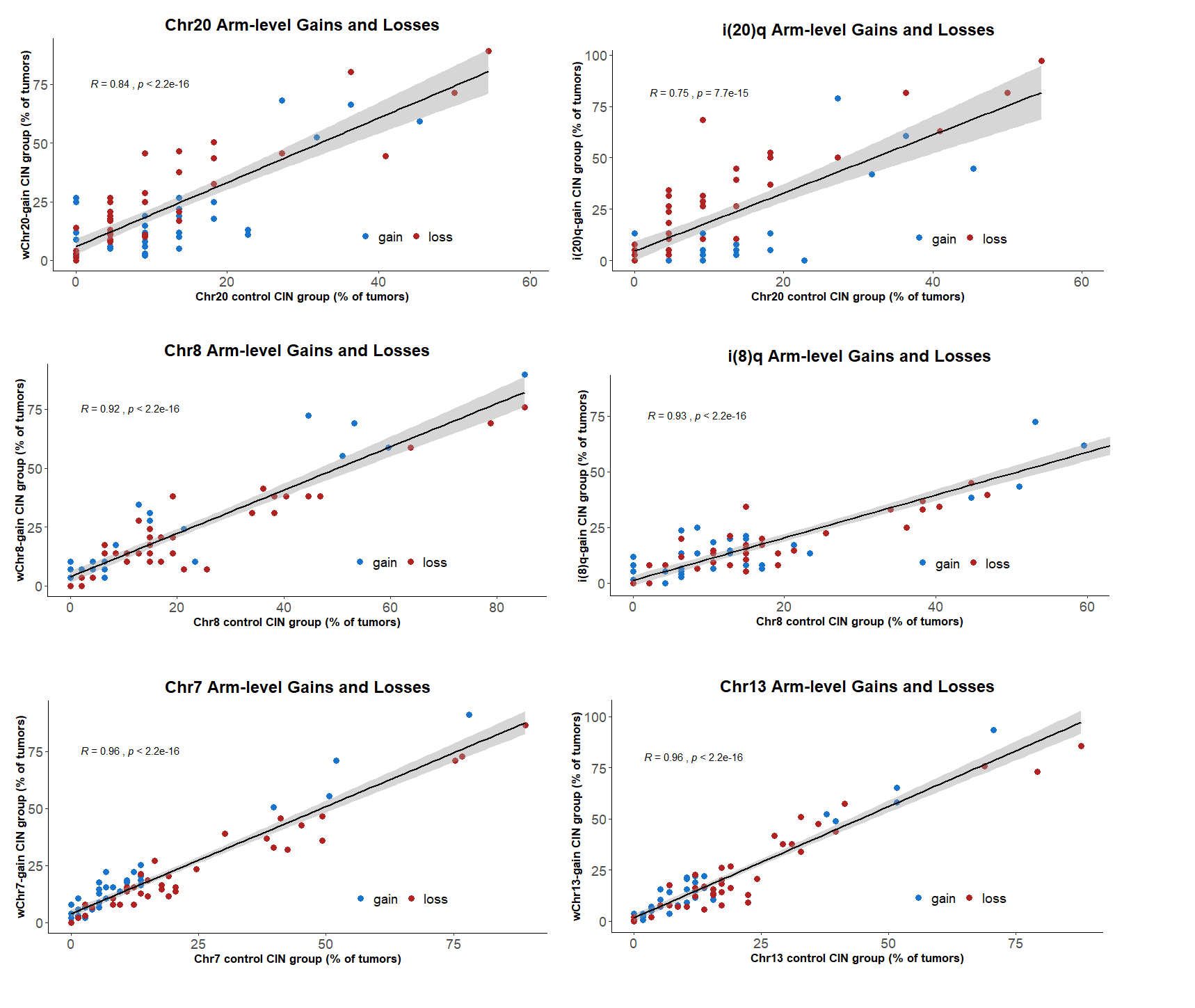

Supplement: Supplementary file 1 [file ijms-20-04652-s001.zip › new Supplementary Figure 1.docx]
