# Supplementary material for: Chromosomal Density of Cancer Up-Regulated Genes, Aberrant Enhancer Activity and Cancer Fitness Genes Are Associated with Transcriptional Cis-Effects of Broad Copy Number Gains in Colorectal Cancer
Source: Int J Mol Sci. 2019 Sep 19;20(18):4652. doi: 10.3390/ijms20184652 (PMC6770609; doi:10.3390/ijms20184652)
Supplement: Supplementary file 1 [file ijms-20-04652-s001.zip › Supplementary Fig 3.docx]

| **wChr8 gain group** | **i(8q) group** |
| --- | --- |
| 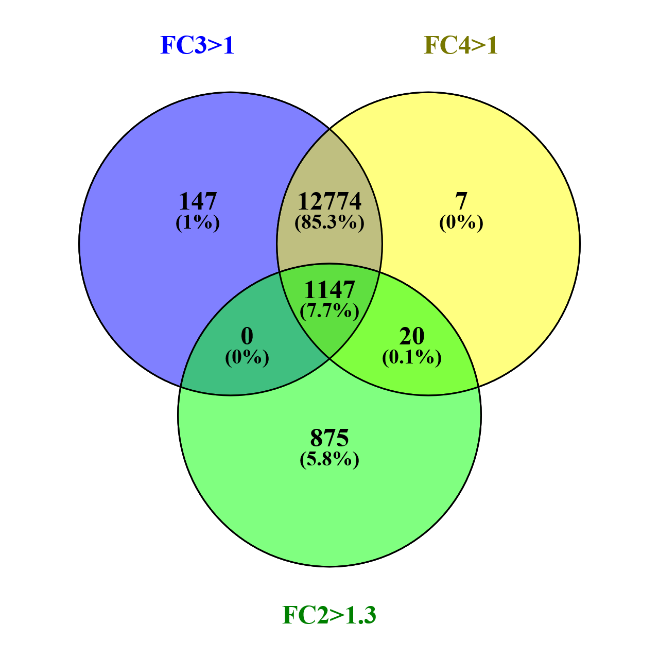 | 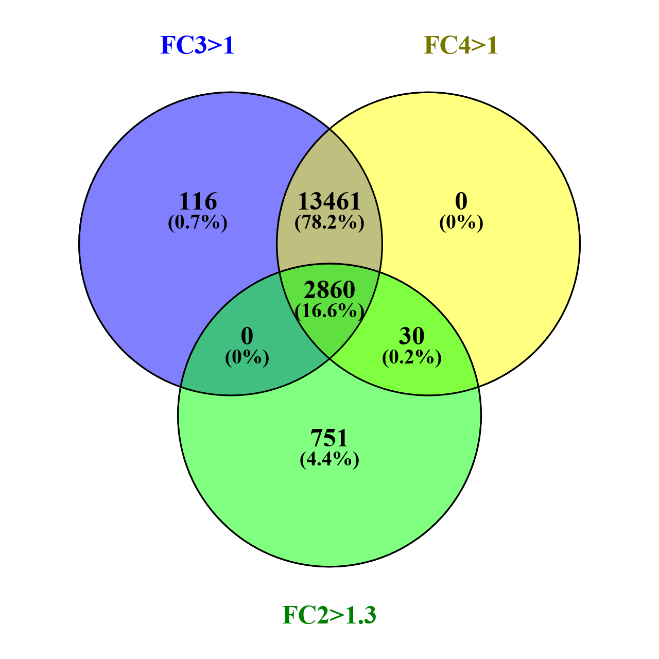 |
| **wChr20 gain group** | **i(20q) group** |
| 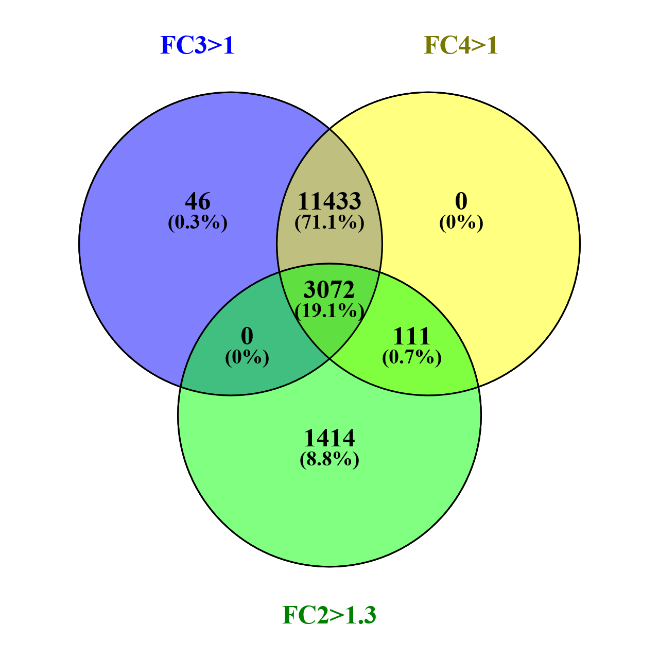 | 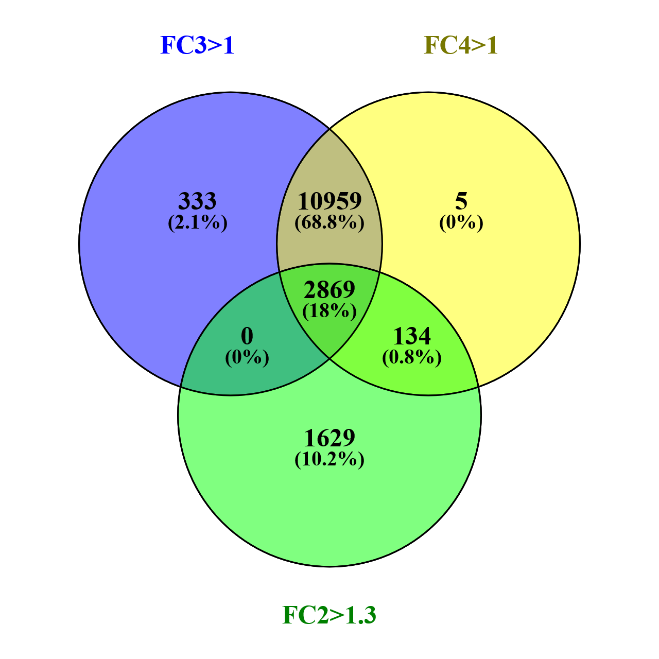 |
| **wChr7 gain group** | **wChr13 gain group** |
| 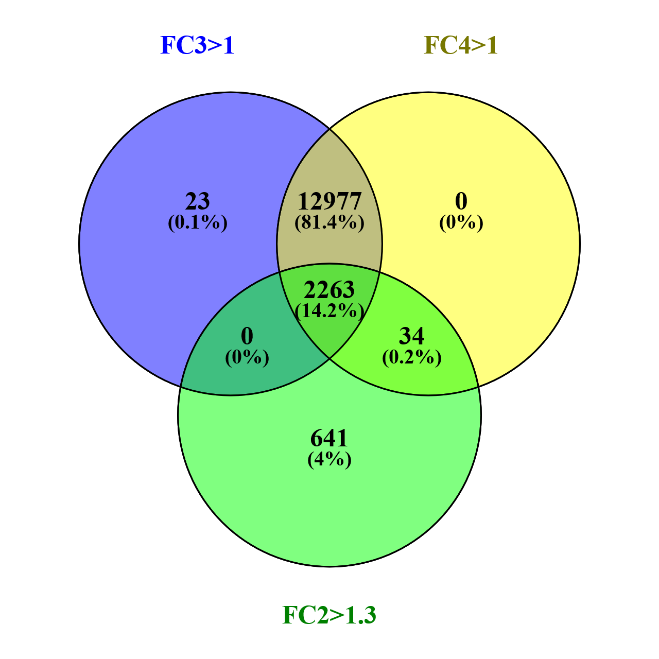 | 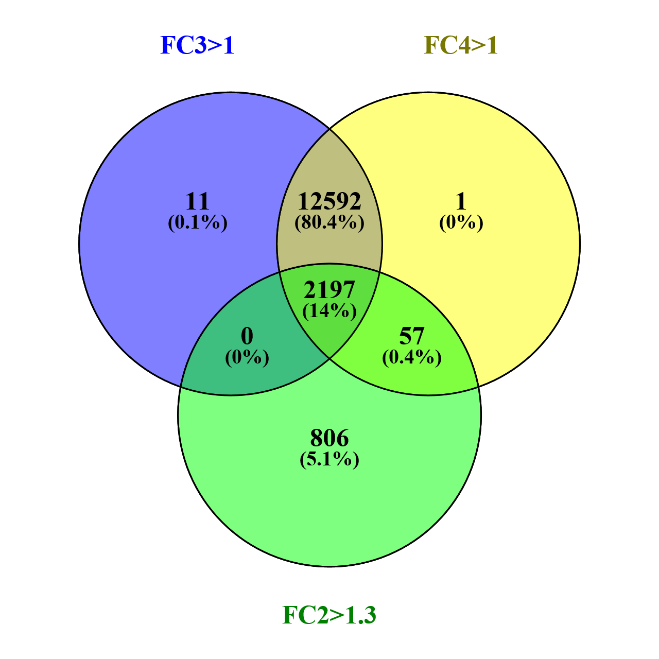 |

| **wChr8 gain CIN group** | **i(8q) CIN group** |
| --- | --- |
| 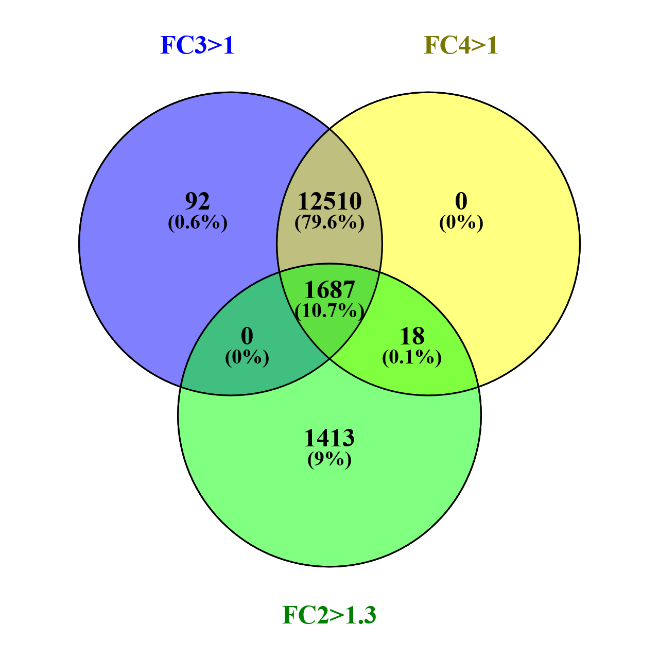 | 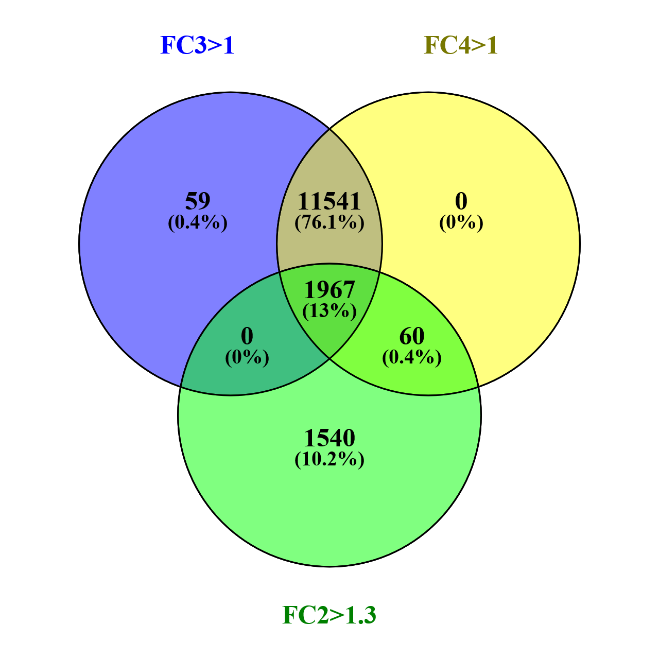 |
| **wChr20 gain CIN group** | **i(20q) CIN group** |
| 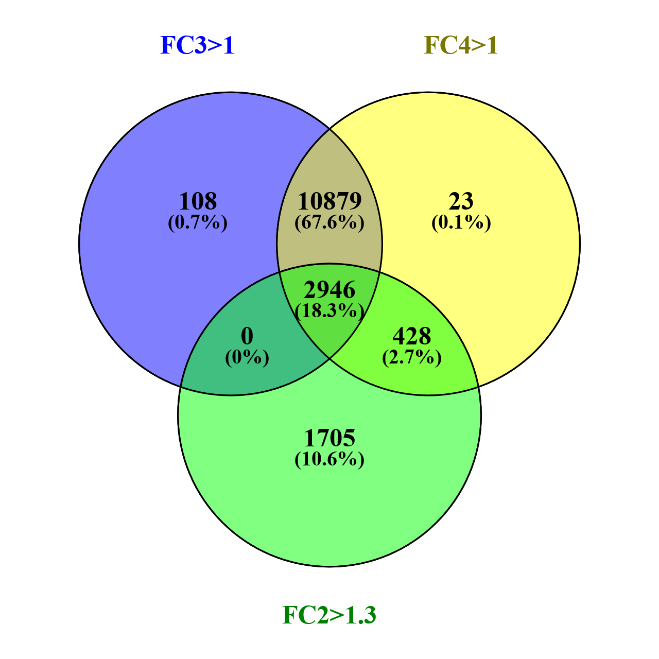 | 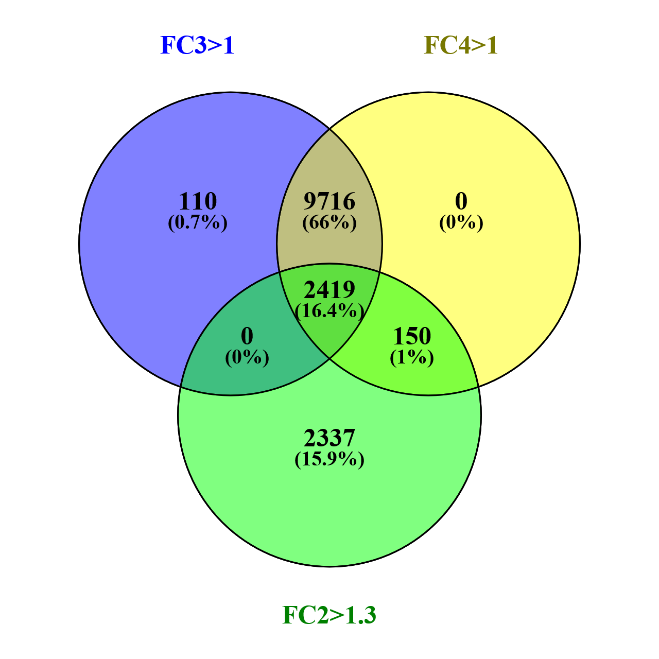 |
| **wChr7 gain CIN group** | **wChr13 gain CIN group** |
| 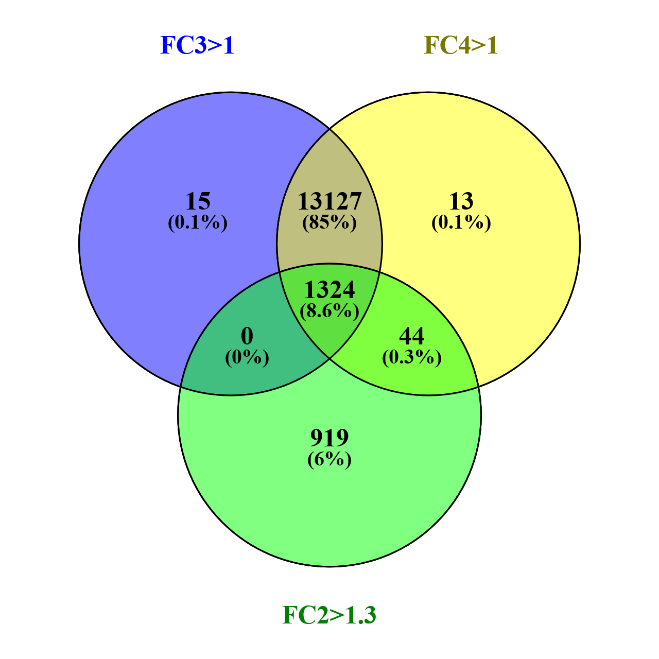 | 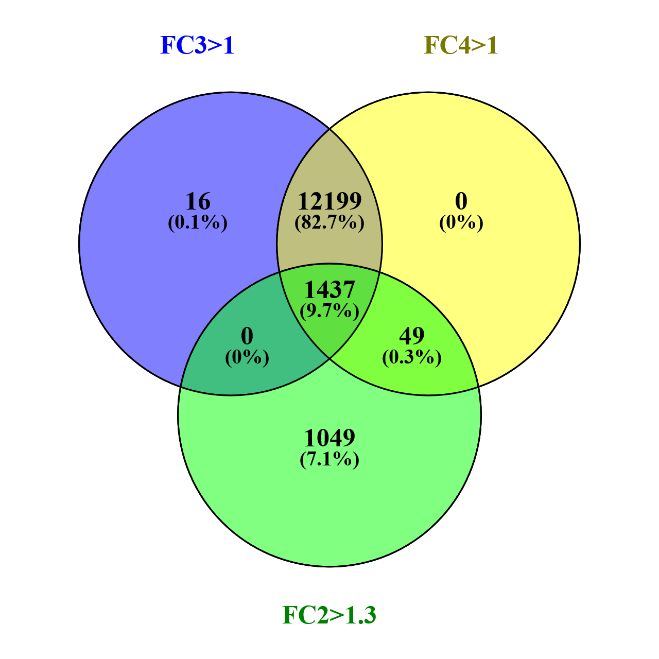 |
