# Supplementary figures and images for: Chromosomal Density of Cancer Up-Regulated Genes, Aberrant Enhancer Activity and Cancer Fitness Genes Are Associated with Transcriptional Cis-Effects of Broad Copy Number Gains in Colorectal Cancer
Source: Int J Mol Sci. 2019 Sep 19;20(18):4652. doi: 10.3390/ijms20184652 (PMC6770609; doi:10.3390/ijms20184652)

**Supplementary Figure 2**


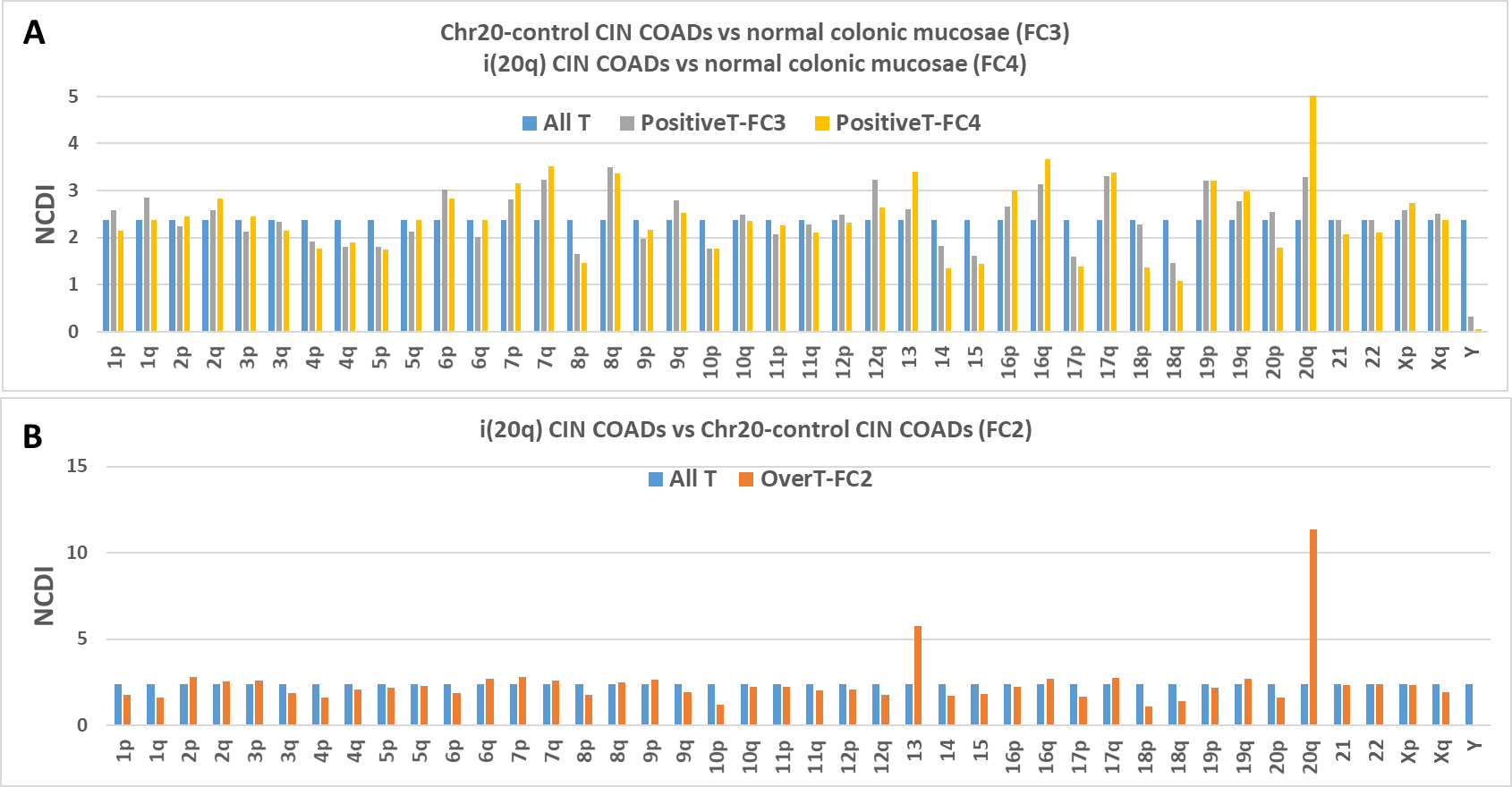


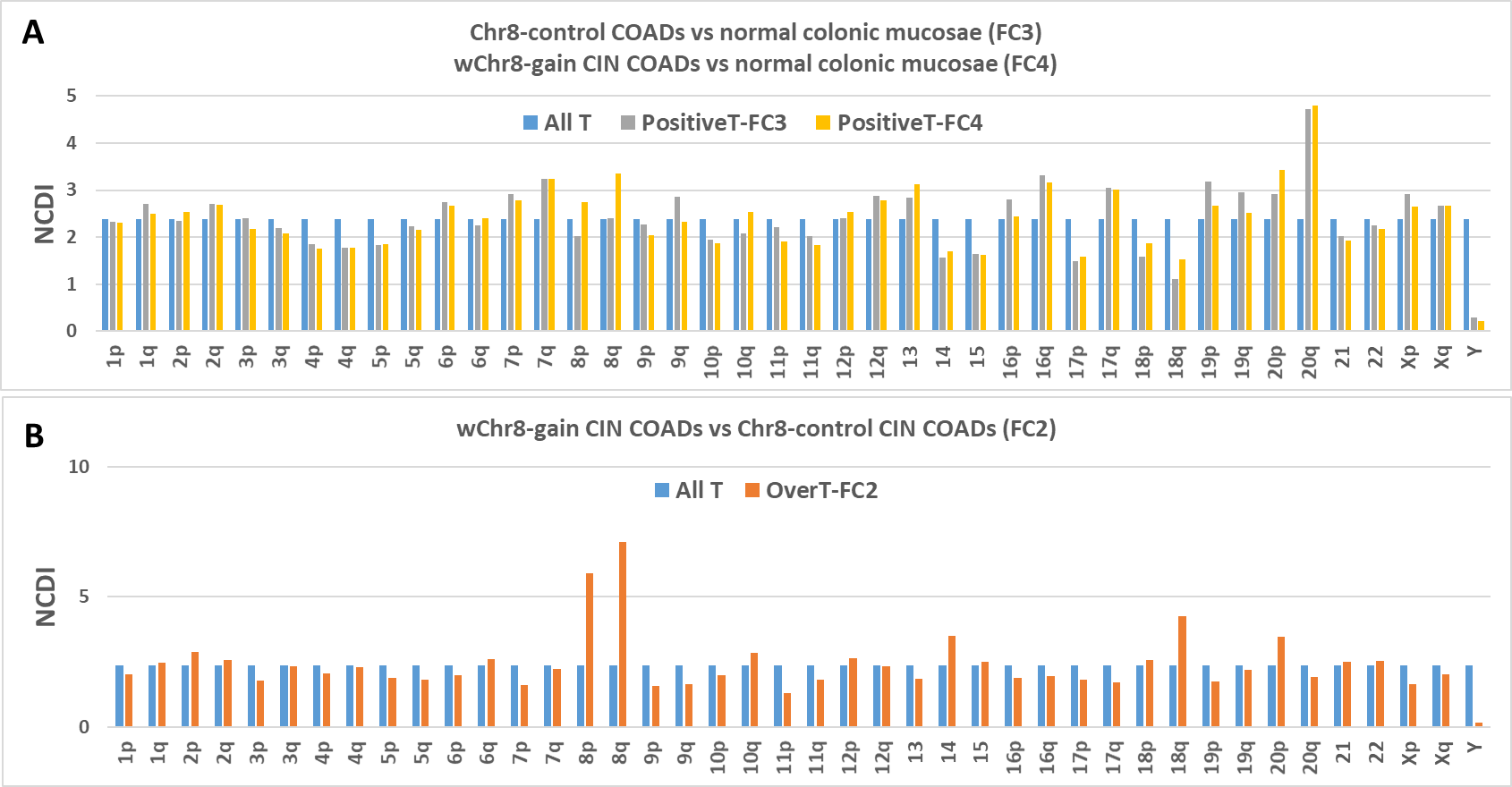


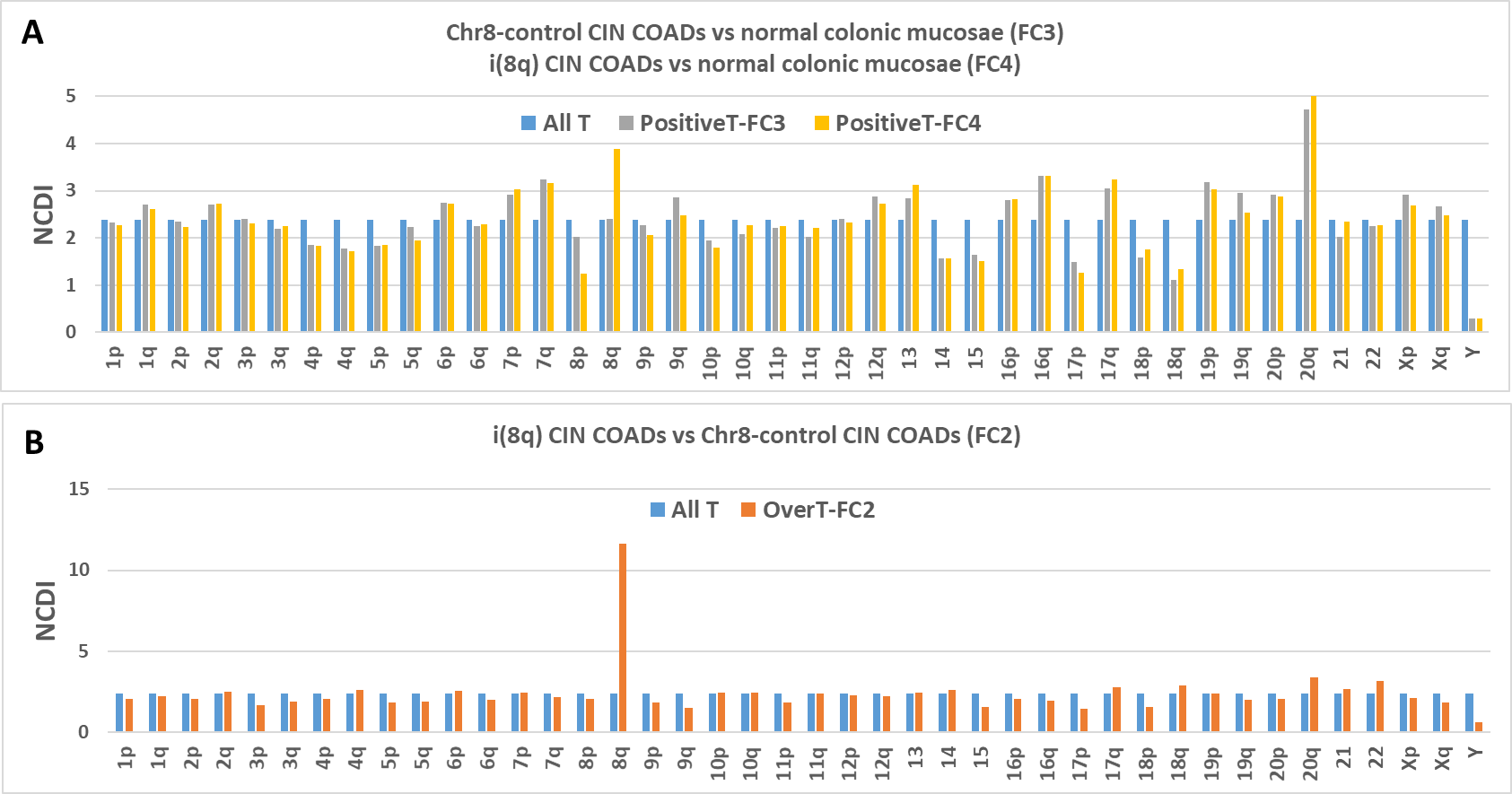


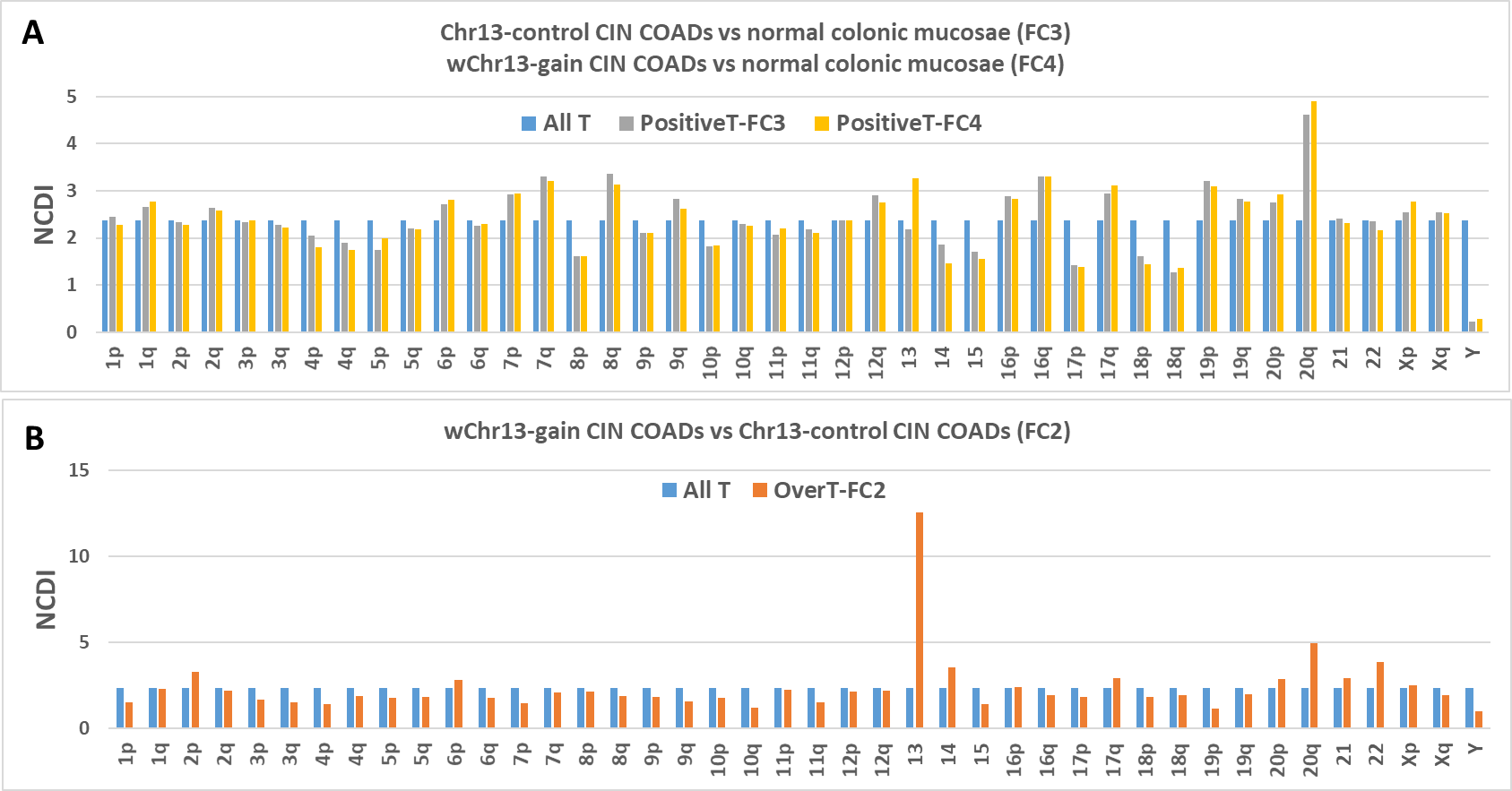


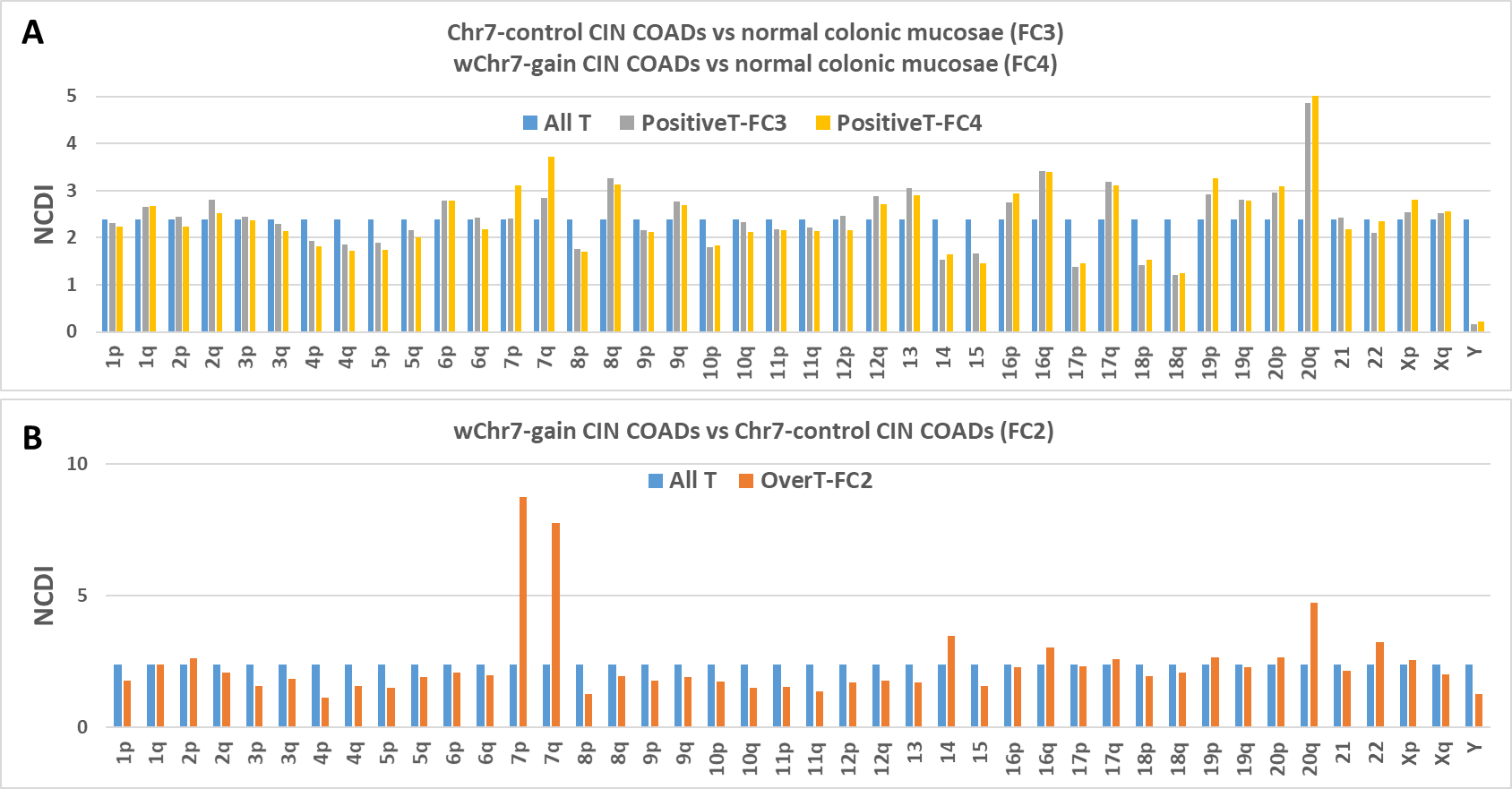

Supplement: Supplementary file 1 [file ijms-20-04652-s001.zip › new Supplementary Figure 2.docx]

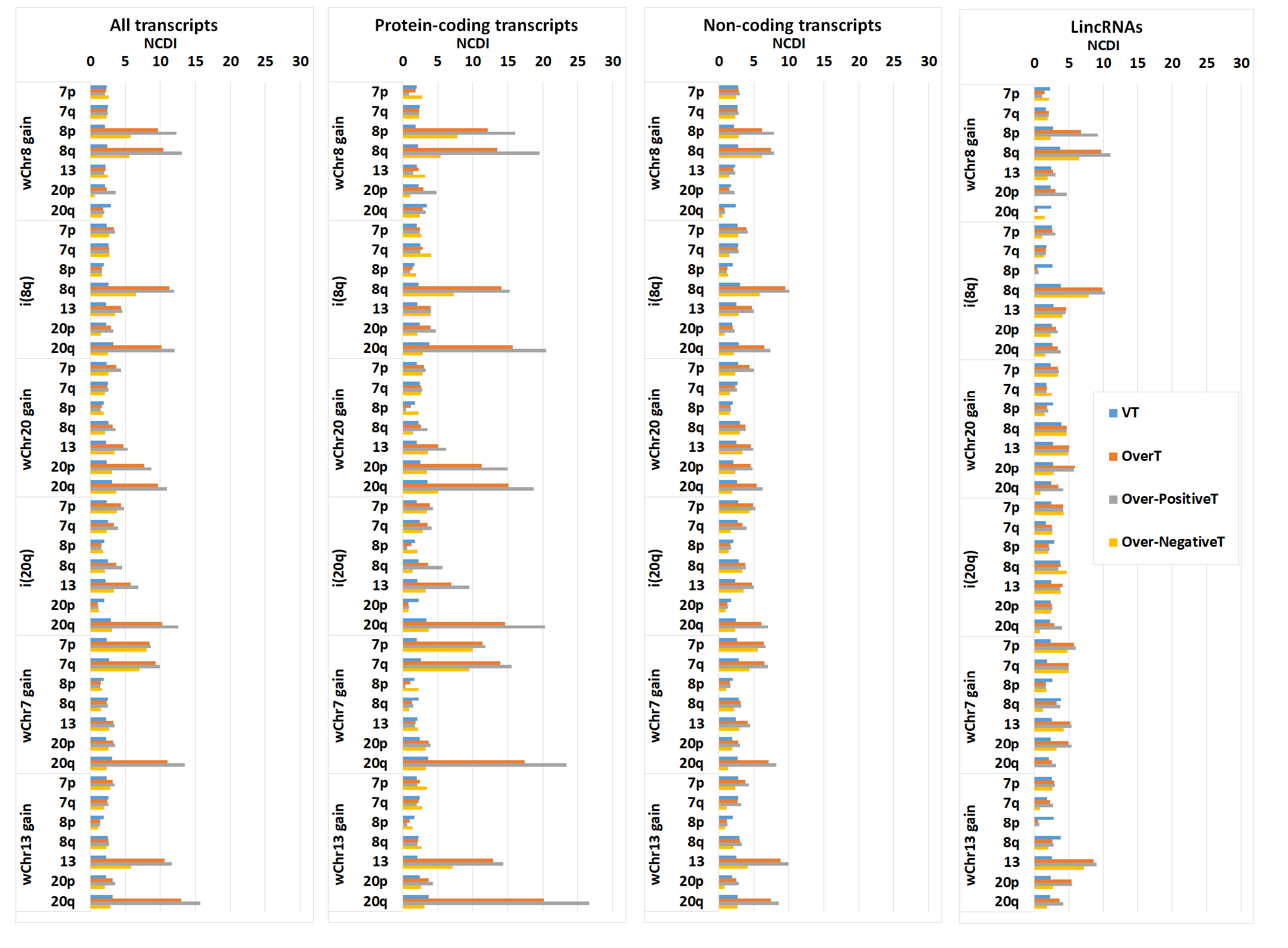

Supplement: Supplementary file 1 [file ijms-20-04652-s001.zip › supplementary Fig 4.tif]

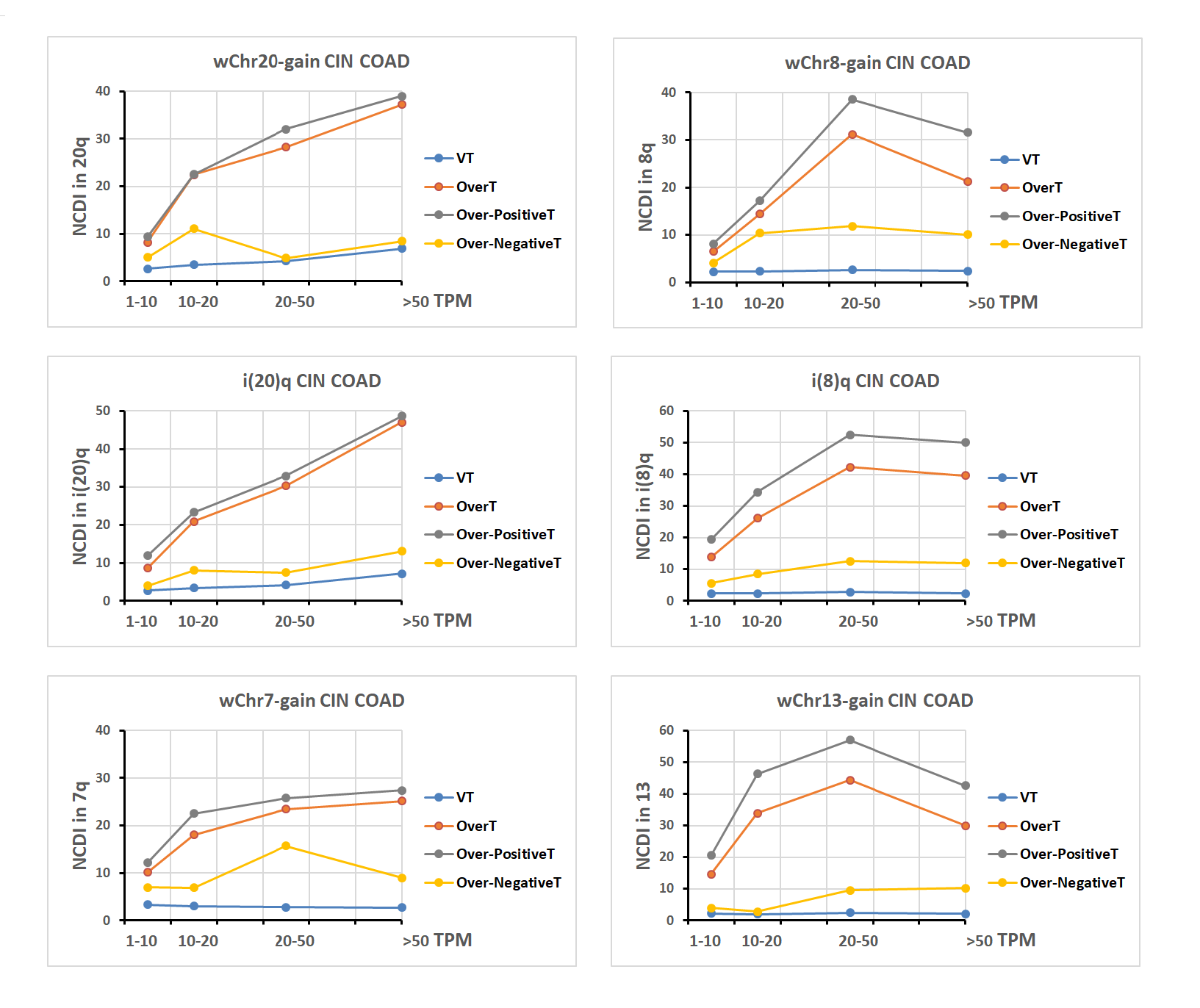

Supplement: Supplementary file 1 [file ijms-20-04652-s001.zip › supplementary Fig 5.tif]

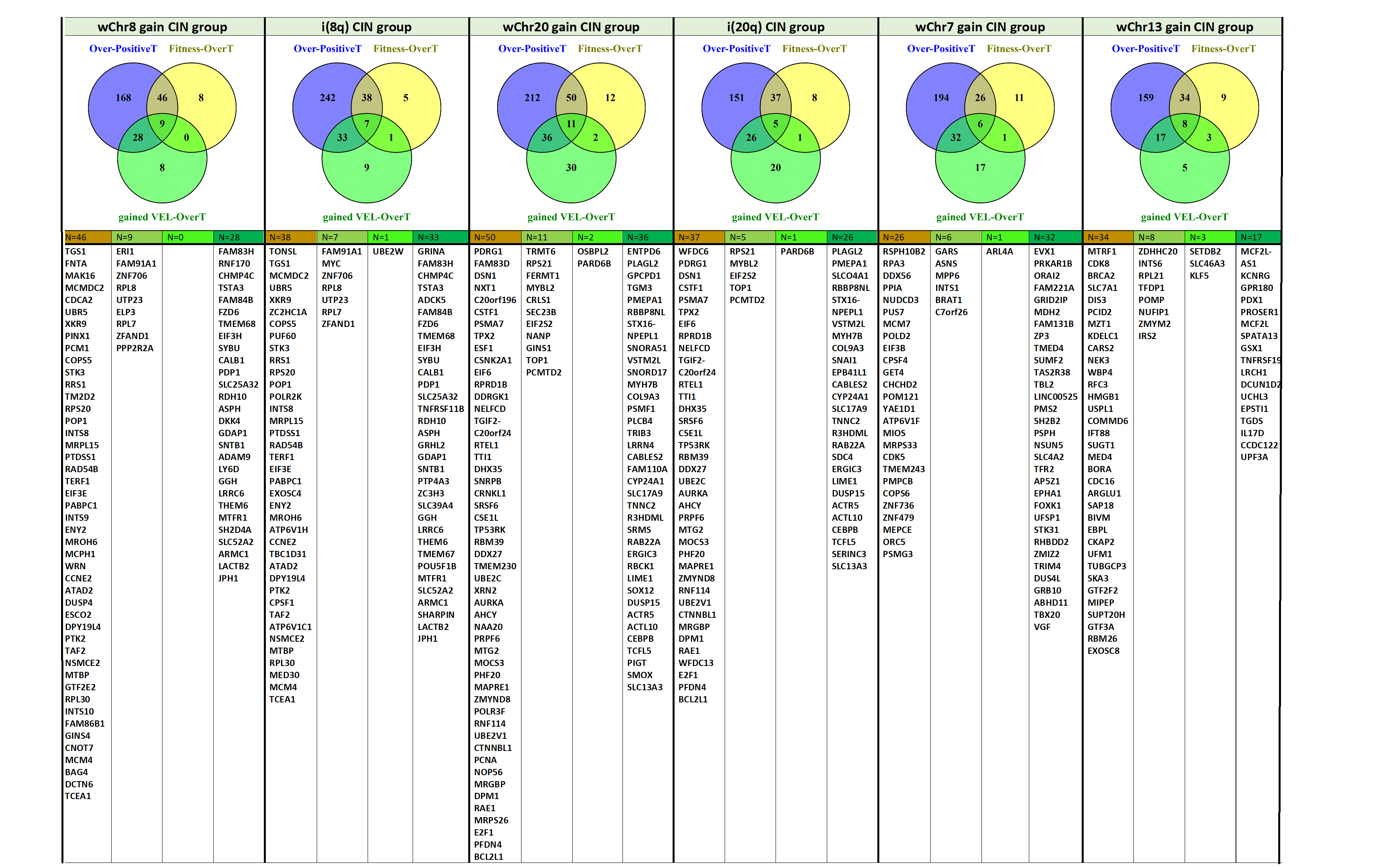

Supplement: Supplementary file 1 [file ijms-20-04652-s001.zip › supplementary Fig 6.tif]
